# Genomic-Led Discovery of a Novel Glycopeptide Antibiotic by *Nonomuraea coxensis* DSM 45129

**DOI:** 10.1021/acschembio.1c00170

**Published:** 2021-04-29

**Authors:** Oleksandr Yushchuk, Natalia M. Vior, Andres Andreo-Vidal, Francesca Berini, Christian Rückert, Tobias Busche, Elisa Binda, Jörn Kalinowski, Andrew W. Truman, Flavia Marinelli

**Affiliations:** § Department of Biotechnology and Life Sciences, University of Insubria, via J. H. Dunant 3, 21100 Varese, Italy; † Department of Molecular Microbiology, John Innes Centre, Norwich, NR4 7UH, United Kingdom; ‡ Technology Platform Genomics, CeBiTec, Bielefeld University, Sequenz 1, 33615 Bielefeld, Germany

## Abstract

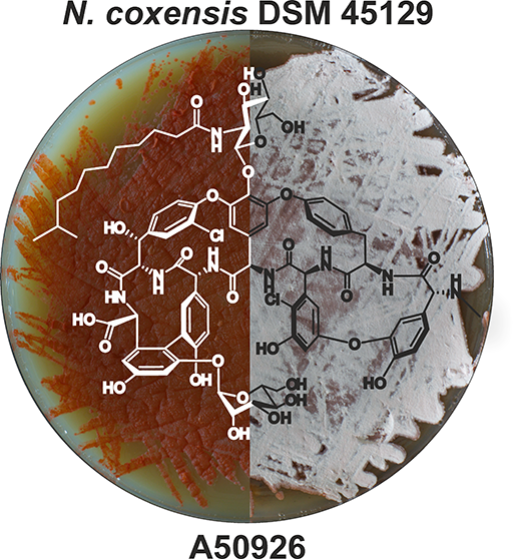

Glycopeptide antibiotics (GPAs) are last defense line drugs against multidrug-resistant Gram-positive pathogens. Natural GPAs teicoplanin and vancomycin, as well as semisynthetic oritavancin, telavancin, and dalbavancin, are currently approved for clinical use. Although these antibiotics remain efficient, emergence of novel GPA-resistant pathogens is a question of time. Therefore, it is important to investigate the natural variety of GPAs coming from so-called “rare” actinobacteria. Herein we describe a novel GPA producer—*Nonomuraea coxensis* DSM 45129. Its *de novo* sequenced and completely assembled genome harbors a biosynthetic gene cluster (BGC) similar to the *dbv* BGC of A40926, the natural precursor to dalbavancin. The strain produces a novel GPA, which we propose is an A40926 analogue lacking the carboxyl group on the *N*-acylglucosamine moiety. This structural difference correlates with the absence of *dbv29*—coding for an enzyme responsible for the oxidation of the *N*-acylglucosamine moiety. Introduction of *dbv29* into *N. coxensis* led to A40926 production in this strain. Finally, we successfully applied *dbv3* and *dbv4* heterologous transcriptional regulators to trigger and improve A50926 production in *N. coxensis*, making them prospective tools for screening other *Nonomuraea* spp. for GPA production. Our work highlights genus *Nonomuraea* as a still untapped source of novel GPAs.

## 1 Introduction

*Nonomuraea* is a genus of so-called “rare” actinomycetes whose potential to produce specialized (secondary) metabolites is still rather poorly explored.^1,2^ Recently sequenced genomes of *Nonomuraea* species appear to be generally larger than the reference *Streptomyces* ones. The mean genome size of *Nonomuraea* (based on the three available complete assemblies^2,3^) is around 12 Mbp, whereas the mean genome size of *Streptomyces* (calculated on 251 fully assembled genomes available in GenBank) equals 8.6 Mbp. The larger genomes of *Nonomuraea* spp. encode dozens of putative biosynthetic gene clusters (BGCs).^2−4^*Nonomuraea* spp. were initially found to be recalcitrant to commonly used genetic engineering manipulations, but new tools are now being developed for this genus.^5−7^ This paves the way for unravelling the huge hidden biosynthetic potential of these organisms.

**Figure 1:**
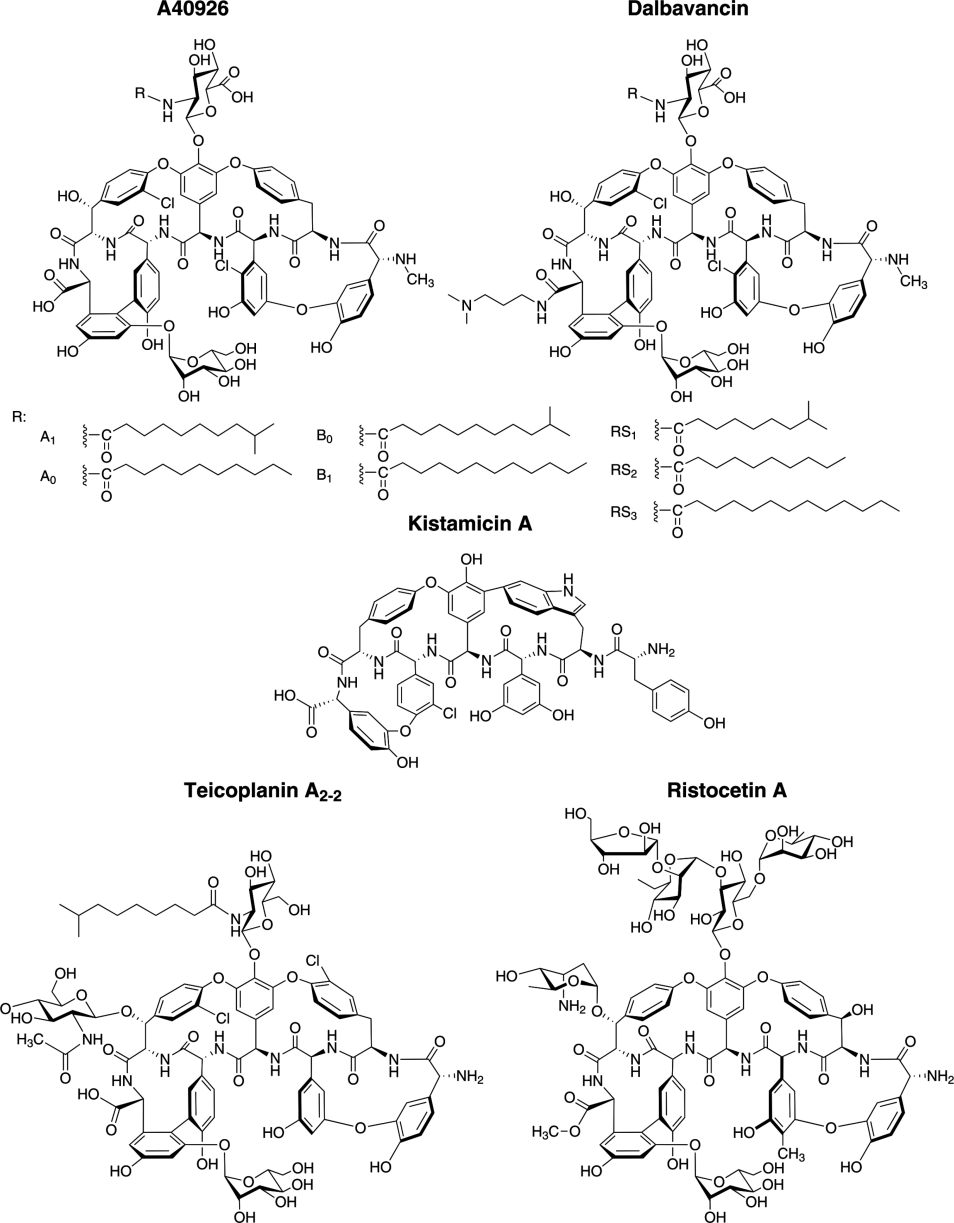
Structures of the GPAs found in genus *Nonomuraea*: type IV A40926 and type V kistamicin. Clinically used dalbavancin is obtained from A40926 by conversion of the C-terminal carboxyl group into a (3-dimethylamino)-1-propylamide. Type IV teicoplanin and type III ristocetin are shown due to their structural similarities with A40926. For teicoplanin, the main factor (TA_2–2_) of the complex produced by *A. teichomyceticus* is shown, the other factors are differing by the length and branching of the lipid chain. Ristocetin is produced by numerous *Amycolatopsis* species.

Probably the most important bioactive metabolite produced by a *Nonomuraea* species is the type IV^8^ glycopeptide antibiotic (GPA) A40926^9^ (Figure 1) produced by *Nonomuraea gerenzanensis* ATCC 39727. Like other GPAs, A40926 acts as a selective and potent inhibitor of cell-wall biosynthesis in Gram-positive bacteria. A40926 is structurally related to the clinically relevant GPA teicoplanin (Figure 1), produced by *Actinoplanes teichomyceticus* ATCC 31121^10,11^ and to ristocetin (Figure 1), previously isolated from numerous *Amycolatopsis* spp. (i.e., *A. lurida* NRRL 2430, *A. japonicum* MG417-CF17, and *Amycolatopsis* sp. MJM2582).^12−14^ Like teicoplanin, A40926 is produced as a mixture of related compounds (major components are A40926 B and A40926 A factors), which differ in the length and branching of an aliphatic side chain (Figure 1). It was recently clarified that *N. gerenzanensis* produces the GPA in the form of *O*-acetyl-A40926 (with an *O*-acetylated mannose residue), but the acetyl group is lost during the alkaline extraction of the antibiotic.^15,16^ Since it was this deacetylated GPA that was initially named A40926, we will refer to it as A40926 hereafter.

A40926 is the precursor of the second-generation semisynthetic GPA dalbavancin (Figure 1), which is currently applied in clinics to treat severe infections caused by multidrug-resistant Gram-positive pathogens.^17^ Dalbavancin (marketed in Europe and USA under the trade names xydalba and dalvance, respectively) is the first antibiotic designated as a qualified infectious disease product by FDA because of its potency, extended dosing interval, and unique dose regimen (once-a-week), but its cost still largely exceeds that of first-generation GPAs.^10^ Therefore, improvement of A40926 production by recombinant engineering of *N. gerenzanensis* has become increasingly relevant.^6,16^ Following the sequencing of the A40926 BGC (*dbv*) almost two decades ago,^18^ multiple aspects of A40926 biosynthesis were investigated, including nonribosomal aglycone assembly and tailoring steps,^15,19,20^ self-resistance,^21,22^ and pathway-specific regulation of its production.^6,23,24^*N. gerenzanensis* was also engineered to produce A40926 derivatives that are better suited for downstream chemical modification to dalbavancin.^16^ Another GPA produced by a *Nonomuraea* species is the type V GPA kistamicin (Figure 1) from *Nonomuraea* sp. ATCC 55076, which was reported to exhibit potent antiviral activity as well as mild antibiosis against Gram-positive bacteria.^2,25^ Its structure contains an unusual indole–phenol cross-link which makes this GPA unique among those already known.^5,26^

Genome mining has recently shown that other species from the genus *Nonomuraea* also possess BGCs for GPAs,^27^ as in the cases of *Nonomuraea* sp. WAC 01424 and *Nonomuraea coxensis* DSM 45129. Notwithstanding the low quality of the available draft genomic data, we recently showed that *N. coxensis* DSM 45129 carries a BGC remarkably similar to *dbv*.^6^ We found that this BGC contains a putative regulatory gene orthologous to *dbv3*, which encodes the pathway-specific regulator of LuxR-type in *N. gerenzanensis*.^6^ The heterologous expression of this gene from *N. coxensis* (named *nocRI*) led to A40926 overproduction in *N. gerenzanensis*, indicating that it might be functional in *N. coxensis* as well. Thus, in this paper we present the fully assembled genome of *N. coxensis*, which has allowed us to properly describe the putative GPA BGC (called *noc*). Additionally, we report that *N. coxensis* produces a novel GPA complex, which we named A50926. Structural characterization of A50926 by liquid chromatography–mass spectrometry (LC-MS) and tandem MS (MS/MS) showed it has high similarity to A40926, although A50926 lacks the carboxyl group on the *N*-acylglucosamine (Glc*N*-Acyl) moiety. Consistently, the *noc* BGC lacks an orthologue of *dbv29*, which in *N. gerenzanensis* encodes the enzyme oxidizing the Glc*N*-Acyl moiety to an *N-*acylaminoglucuronic group.^19^ Introduction of *dbv29* into *N. coxensis* changed the GPA production profile of this strain to A40926. Finally, we have introduced *dbv3* and *dbv4* pathway-specific regulatory genes in *N. coxensis* to trigger and overproduce A50926 by regulatory gene cross-talking. In conclusion, our results describe the biosynthesis of a novel GPA, which may have superior properties to A40926^28^ and thus may contribute to developing a platform for the combinatorial biosynthesis of third generation lipo-GPAs.

## 2 Results and Discussion

### 2.1 Complete Assembly of *N. coxensis* Genome Reveals the Presence of a Novel GPA BGC

The presence of a novel GPA BGC in the genome of *N. coxensis* was recently anticipated.^6,27^ However, due to the poor quality of the available draft, fragments of the BGC were found on different contigs and did not cover the full expected sequence of the BGC. Therefore, we sequenced and fully assembled the genome of *N. coxensis* DSM 45129 using a combination of HiSeq Illumina and GridION ONT technologies. The circular chromosome of *N. coxensis* was found to have a smaller size in comparison to the other two previously published *Nonomuraea* genomes—only 9.07 Mbp compared to 11.85 Mbp in *N. gerenzanensis*(3) and 13.05 Mbp in *Nonomuraea* sp. ATCC 55076.^2^ The average GC-content was 71.8%. Annotation of the *N. coxensis* genome revealed 8398 predicted protein coding sequences, five operons for 16S-23S-5S rRNA, and 73 tRNA genes. Genome analysis by antiSMASH 5.0,^29^ a specialized metabolite BGC identification tool, led to the discovery of 27 putative BGCs when used in the “relaxed” search mode. However, only a few BGCs showed more than 20% similarity to known BGCs (Table S1).

**Figure 2:**
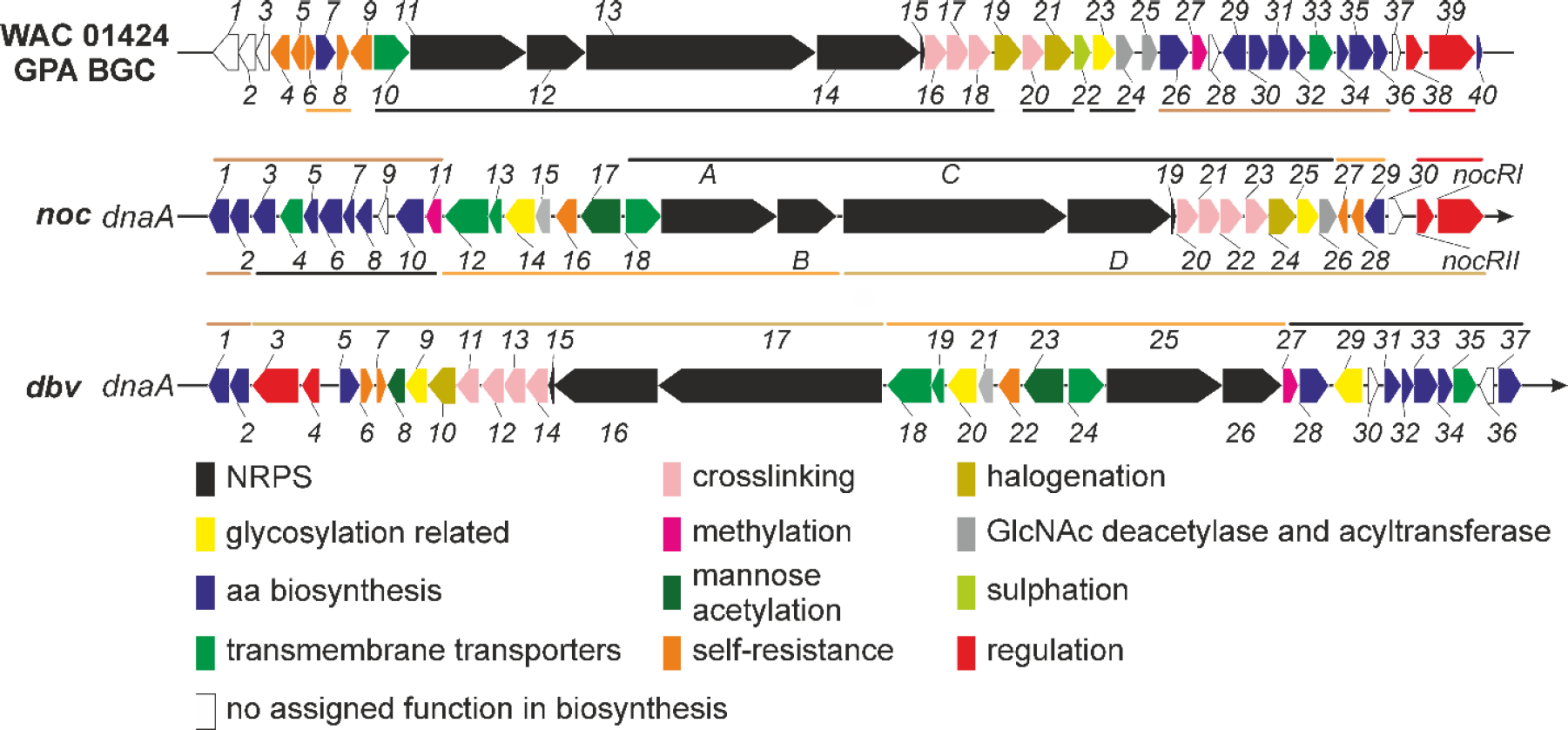
Comparison of BGCs from *N. gerenzanensis* (*dbv*), *N. coxensis* (*noc*), and *Nonomuraea* sp. WAC 01424. Colored lines indicate the homology segments among the BGCs. For *dbv* and *noc*, the orientation of the BGC genes is in relation to the orientation of the *dnaA* gene of the chromosome. This orientation was not possible for the WAC 01424 GPA BGC, since the corresponding genome is fragmented across multiple contigs. Details on gene function and homology are given in Table 1 and in the main text.

We thus focused our attention on the GPA-like BGC, which we denoted as *noc* (from ***No**nomuraea**c**oxensis*). The *noc* BGC is the fourth GPA BGC described from *Nonomuraea* genus, following the *dbv* BGC from *N. gerenzanensis*,^18^ a putative GPA BGC from *Nonomuraea* sp. WAC 01424^27^ and the type V GPA kistamicin (*kis*) BGC from *Nonomuraea* sp. ATCC 55076.^2^ Overall, *noc* contains 36 open reading frames (ORFs) with 35 among them homologous to *dbv* genes (the nonhomologous *noc* gene encoding for a putative transposase) and 32 being homologous to genes in the *Nonomuraea* sp. WAC 01424 GPA BGC (Figure 2, Table 1). The *kis* BGC differed from *noc* most significantly (data not shown).

**Table 1:** Characterization of *noc* BGC genes and their comparison to the *dbv* and WAC 01424 GPA BGCs from *Nonomuraea* spp.

*noc* BGC genes	homologues from *dbv* BGC (aa identity of protein product with *noc* homologue, %)	homologues from WAC 01424 GPA BGC (numbered as in Figure 2) (aa identity of protein product with *noc* homologue, %)	encoded protein
*noc1*	*dbv1* (90.6%)	*DMB42_RS42735* (31) (60%)	hydroxymandelate oxidase (Hmo)
*noc2*	*dbv2* (89.3%)	*DMB42_RS42740* (30) (62%)	hydroxymandelate synthase (HmaS)
*noc3*	*dbv37* (90.9%)	*DMB42_RS42745* (29) (83%)	hydroxyphenylglycine aminotransferase (HpgT)
*noc4*	*dbv35* (90.9%)	*DMB42_RS42730* (32) (63%)	Na^+^–H^+^ antiporter
*noc5*	*dbv34* (93.9%)	*DMB42_RS42710* (36) (87%)	enoyl-CoA hydratase (DpgD)
*noc6*	*dbv33* (89.2%)	*DMB42_RS42715* (35) (84%)	dihydroxyphenylacetyl-CoA dioxygenase (DpgC)
*noc7*	*dbv32* (85.1%)	*DMB42_RS42720* (34) (74%)	enoyl-CoA hydratase (DpgB)
*noc8*	*dbv31* (94.3%)	*DMB42_RS42725* (33) (91%)	type III polyketide synthase (DpgA)
*noc9*	*dbv30* (83.5%)	*DMB42_RS42750* (28) (69%)	4HB-CoA thioesterase
*noc10*	*dbv28* (92.4%)	*DMB42_RS42760* (26) (86%)	β-hydroxylase
*noc11*	*dbv27* (91.8%)	*DMB42_RS42755* (27) (58%)	methyltransferase
*noc12*	*dbv18* (87.3%)	a	ABC transporter
*noc13*	*dbv19* (92.2%)	a	ABC transporter
*noc14*	*dbv20* (89.7%)	a	mannosyltransferase
*noc15*	*dbv21* (86.6%)	*DMB42_RS*42765 (25) (64%)	deacetylase
*noc16*	*dbv22* (92.3%)	*DMB42_RS*42850 (9) (77%)	sensory histidine kinase
*noc17*	*dbv23* (88.1%)	a	acetyltransferase
*noc18*	*dbv24* (92.4%)	*DMB42_RS42845* (10) (81%)	ABC transporter
*nocA*	*dbv25* (88.7%)	*DMB42_RS42840* (11) (76%)	NRPS modules 1–2
*nocB*	*dbv26* (91%)	*DMB42_RS42835* (12) (78%)	NRPS module 3
*nocC*	*dbv17* (89.6%)	*DMB42_RS42830* (13) (77%)	NRPS modules 4–5–6
*nocD*	*dbv16* (91.7%)	*DMB42_RS42825* (14) (79%)	NRPS module 7
*noc19*	*dbv15* (94.2%)	*DMB42_RS42820* (15) (93%)	MbtH-like protein
*noc20*	*dbv14* (91.8%)	*DMB42_RS42815* (16) (78%)	cross-linking oxygenase (OxyA)
*noc21*	*dbv13* (89.8%)	*DMB42_RS42810* (17) (77%)	cross-linking oxygenase (OxyC)
*noc22*	*dbv12* (93.5%)	*DMB42_RS42805* (18) (77%)	cross-linking oxygenase (OxyB)
*noc23*	*dbv11* (91.9%)	*DMB42_RS42795* (20) (78%)	cross-linking oxygenase (OxyE)
*noc24*	*dbv10* (94.1%)	*DMB42_RS42790* (21) (87%)	halogenase
*noc25*	*dbv9* (90.4%)	*DMB42_RS42780* (23) (74%)	glycosyltransferase (GtfB)
*noc26*	*dbv8* (87.5%)	*DMB42_RS42775* (24) (77%)	acyltransferase
*noc27*	*dbv7* (87.3%)	*DMB42_RS42865* (6) (78%)	VanY-carboxypeptidase
*noc28*	*dbv6* (95.9%)	*DMB42_RS42855* (8) (92%)	response regulator
*noc29*	*dbv5* (92.8%)	*DMB42_RS42860* (7) (85%)	prephenate dehydrogenase (Pdh)
*noc30*	a	a	putative transposase
*nocRII*	*dbv4* (94.4%)	*DMB42_RS42700* (38) (85%)	StrR-like transcriptional regulator
*nocRI*	*dbv3* (86.3%)	*DMB42_RS42695* (39) (70%)	LuxR-like transcriptional regulator

a Homologue is absent.

### 2.2 Comparative Genomics of *Nonomuraea* GPA Producers

At the time of writing, genomic information for 34 *Nonomuraea* species was available in GenBank, although there are only three complete assemblies (Table S2). Along with the four reported *Nonomuraea* GPA BGCs, we found a *kis*-like BGC in the draft genome of *Nonomuraea* sp. NN258 (Figure S1). We have then reconstructed the multilocus phylogeny (MLP) of all *Nonomuraea* species with available genomic data using conserved house-keeping proteins (Table S3). It revealed *N. coxensis* to be most closely related to *N. wenchangensis* CGMCC 4.5598, *N. polychroma* DSM 43925, and *N. turkmeniaca* DSM 43926 (Figure S2). None of these species have GPA BGCs in their genomes. *N. gerenzanensis* is most closely related to *Nonomuraea* sp. FMUSA5–5 and to the kistamicin producer *Nonomuraea* sp. ATCC 55076, whereas *Nonomuraea* sp. WAC 01424 is distantly related to both *N. coxensis* and *N. gerenzanensis* (Figure S2). Thus, GPA-producing *Nonomuraea* species do not form a single phylogenetic group, which is different from what occurs in the majority of *Amycolatopsis* spp. producing GPAs.^30^

Since *N. gerenzanensis* and *Nonomuraea* sp. ATCC 55076 are closely related and their genomes had been completely assembled, we compared their sequences using the MAUVE genome alignment tool.^31^ We found that the two genomes are very similar, having few rearranged homologous segments (Figure S3A). Interestingly, the regions flanking the *dbv* BGC in *N. gerenzanensis* show synteny in *Nonomuraea* sp. ATCC 55076, but in this genome, they flank a miscellaneous assemblage of GPA-unrelated genes instead of the *dbv* genes. No *dbv*-like BGC is present in *Nonomuraea* sp. ATCC 55076. Similarly, no *kis*-like BGC is in the *N. gerenzanensis* genome, but the regions flanking the *kis* BGC in *Nonomuraea* sp. ATCC 55076 have their homologous counterparts in the *N. gerenzanensis* genome (Figure S3A). Dot plots of *N. gerenzanensis* and *Nonomuraea* sp. ATCC 55076 confirm the high homology between the two strains (Figure S3B). A possible explanation is that *Nonomuraea* sp. ATCC 55076 and *N. gerenzanensis* genomes might have acquired different GPA BGCs independently through horizontal gene transfer (HGT) events from other *Nonomuraea* (or not) species.

Dot plots of *N. coxensis* and *Nonomuraea* sp. ATCC 55076 genomes (Figure S3C) as well as of *N. coxensis* and *N. gerenzanensis* (Figure S3D) indicate that *N. coxensis* is more distantly related to the other GPA producing species. Unfortunately, it was impossible to compare the genome of *N. coxensis* with its closest relatives *N. wenchangensis* CGMCC 4.5598, *N. polychroma* DSM 43925, and *N. turkmeniaca* DSM 43926 (Figure S2), due to the incompleteness of their genome assemblies. Overall, it seems that the position of GPA BGCs is not conserved within *Nonomuraea* genomes, which contrasts to what was observed in most *Amycolatopsis* spp.^30^

### 2.3 Comparing *noc* and *dbv* Biosynthetic Pathways: From Genes to Products

**Figure 3:**
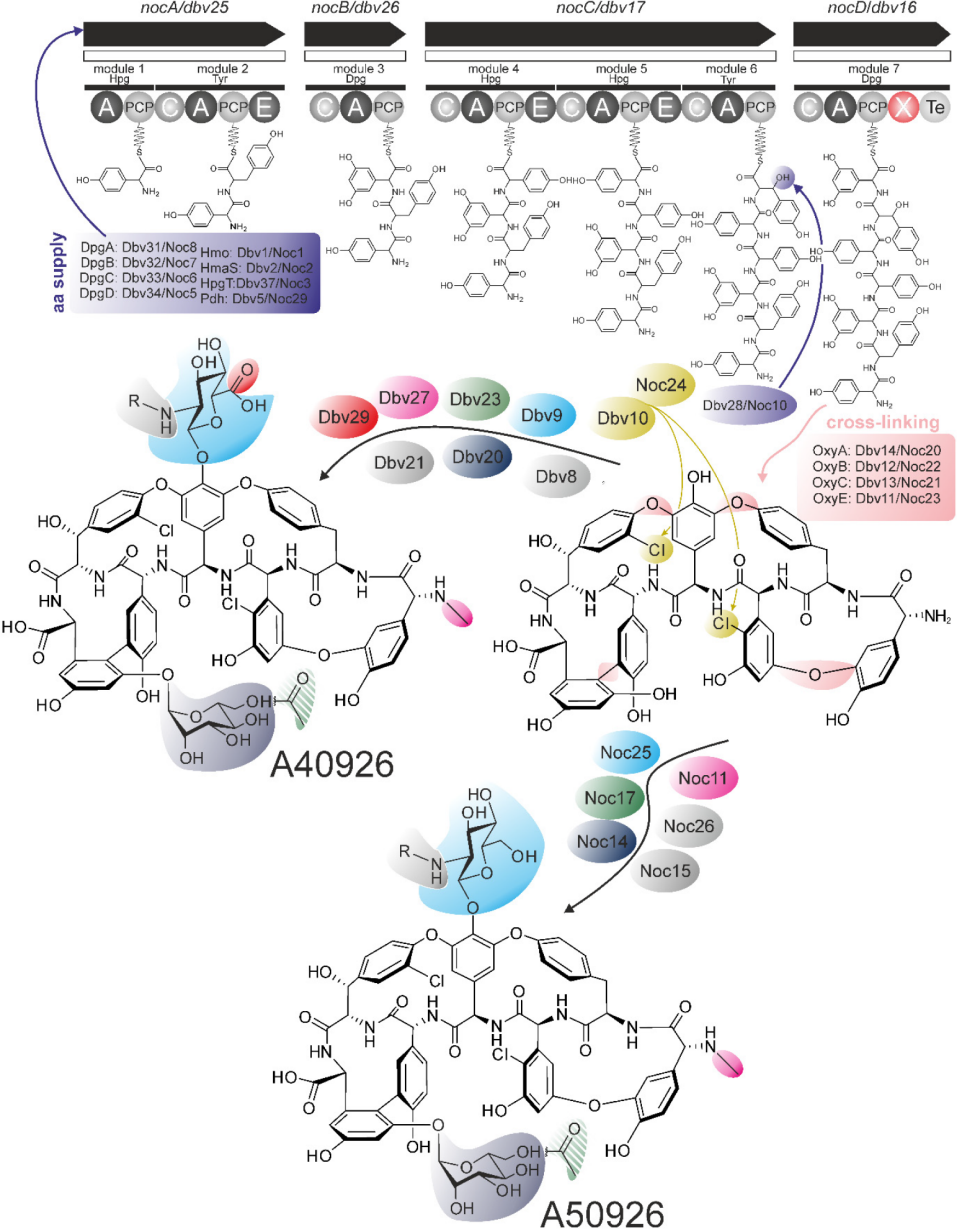
Conceptual scheme of the biosynthesis of A40926 and of the GPA (named A50926) from *N. coxensis*. Please note the dashed acyl group at the mannose residue, which is installed by Dbv23/Noc17 but consequentially lost during antibiotic extraction. For more details and encoded protein names, please refer to the main text and Table 1.

The biosynthesis of A40926 is well understood (Figure 3). The heptapeptide core of this antibiotic is synthesized by a nonribosomal peptide synthetase (NRPS) assembly line involving Dbv25, Dbv26, Dbv17, and Dbv16 proteins. The linear peptide is cross-linked by four monooxygenases (Dbv14, Dbv12, Dbv13, and Dbv11) and halogenated by Dbv10, giving the core aglycone. This aglycone is further modified with the glycosyltransferases Dbv9 and Dbv20, which attach *N-*acetyl glucosamine (Glc*N*Ac) and mannose, respectively.^32^ Then, Glc*N*Ac is oxidized by Dbv29, deacetylated by Dbv21, and acylated by Dbv8. Finally, the mannose moiety is acetylated by Dbv23, giving *O*-acetyl-A40926.

Considering the A40926 pathway, it was possible to predict the biosynthetic pathway of the putative GPA from *N. coxensis* (Figure 3). Sets of genes required for the biosynthesis of the nonproteinogenic precursor amino acids 4-hydroxyphenylglycine (Hpg), 3,5-dihydroxyphenylglycine (Dpg), and β-hydroxytyrosine (further used as substrates for NRPS) are the same in *noc* and *dbv* BGCs (Table 1, Figures 2 and 3). Next, the NRPS, encoded within *noc* BGC, was found to have the same organization and A-domain specificities as the *dbv* NRPS (Figure S4, Table S4). All other genes, responsible for the cross-linking and tailoring steps, were identical in both the *noc* and *dbv* pathways (Table 1, Figures 2 and 3). However, one notable difference between *dbv* and *noc* was the absence of a *dbv29* orthologue in the latter. As mentioned above, Dbv29 is a hexose oxidase responsible for the oxidation of the Glc*N*-Acyl moiety of A40926.^19^ On this basis, we predicted that the *noc* pathway might produce an A40926 analogue lacking the carboxylic group on the Glc*N*-Acyl residue and therefore resembling teicoplanin in this moiety (Figures 1 and 3).

Beyond the biosynthetic genes, *noc* and *dbv* feature homologous regulatory genes. Two master regulators of A40926 biosynthesis—LuxR-like Dbv3 and StrR-like Dbv4—have orthologues coded within *noc*—NocRI (94% aa sequence identity) and NocRII (86% aa sequence identity), respectively.^6^ In *N. gerenzanensis*, both Dbv3 and Dbv4 are crucial for biosynthesis activation.^23^ Dbv4 was shown to bind the promoter regions of operons *dbv30-35* (mainly coding for Dpg biosynthesis enzymes) and *dbv14-8* (including the genes coding for cross-linking monooxygenases), and its binding sites were identified.^33^ Our *in silico* analysis indicates that identical binding sites are present in the promoter regions of *noc20* and *noc8*, orthologues of *dbv14* and *dbv30*, respectively (Figure S5). DNA-binding sites of Dbv3 remain uncharacterized, but its regulon was defined from gene expression analysis and includes other biosynthetic genes and Dbv4.^23^ Given all these similarities, we presume that NocRI/NocRII have functions identical to Dbv3/Dbv4 and both regulatory pairs might cross-talk between these species. Our previous results,^6^ where heterologous expression of *nocRI* in *N. gerenzanensis* improved A40926 production, support this assumption. The single GPA resistance determinant encoded within *noc* is Noc27, a close (87%) orthologue of Dbv7 (VanY_n_), which is a d,d-carboxypeptidase involved in A40926 self-resistance.^21,34^

Although the biosynthetic, regulatory, and resistance genes are apparently shared by the *dbv* and *noc* BGCs, their genetic organization is different. So far, almost all GPA BGCs have NRPS genes located on one strand in an order that is colinear to the order of the modules in the NRPS assembly line. The only exception is the *dbv* BGC, where the NRPS genes are coded on different strands and are separated by other biosynthetic genes.^18^ The *noc* BGC, although sharing a remarkable similarity with *dbv*, features an organization of NRPS genes that is typical of all the other GPAs. Interestingly, only two chromosomal inversion events are needed to rearrange *noc* into *dbv* (Figure S6), indicating how a *dbv-*like gene arrangement might have derived from a *noc-*like BGC in a common ancestor of *N. coxensis* and *N. gerenzanensis* (or in an ancestralprotocluster).

The putative GPA BGC in *Nonomuraea* sp. WAC 01424 (Figure 2) differs more substantially from both *noc* and *dbv.* It lacks a *noc14/dbv20* homologue encoding for a mannosyltransferase, as well as a *noc17/dbv23* homologue encoding for a mannose-*O-*acetyltransferase (Table 1). Instead, WAC 01424 GPA BGC contains a close homologue of *staL* (Figure S7), which encodes for a sulfotransferase involved in the biosynthesis of A47934 from *Streptomyces toyocaensis* NRRL 15009.^35^ Additionally, the WAC 01424 BGC-encoded halogenases seem more related to the ones from the A47934 BGC than to Noc24 and Dbv8 (Figure S7). Thus, we suggest that WAC 01424 GPA is a nonmannosylated, but sulfated, A40926 analogue, putatively with a halogenation pattern different from A40926 (Figure S8).

### 2.4 Optimization of GPA-Producing Conditions for *N. coxensis*

*N. coxensis* was first described in 2007,^36^ but as far as we know, it was never tested for the production of antimicrobials. Considering the predicted similarity between the putative GPA produced by *N. coxensis* with A40926, we first applied to *N. coxensis* the cultivation and A40926 production conditions that we had previously optimized for *N. gerenzanensis*.^22,37^ In these conditions (namely a vegetative preculture in E26 medium and a GPA production step in FM2 medium using baffled flasks), *N. coxensis* tended to grow poorly, and no antimicrobial activity was detectable throughout the 168h cultivation from inoculum. Thus, we further screened different media and fermentation conditions previously used for growing other GPA producing strains, such as TM1 used for teicoplanin production by *A. teichomyceticus*(38) and R5 adopted for balhimycin production in *Amycolatopsis balhimycina*,^39^ as well as VM0.1 and ISP2l previously employed for the vegetative cultivation of *N. coxensis*(6) (media composition detailed in the Supporting Information). The production of antimicrobial activity toward *Bacillus subtilis* ATCC 6633 was observed only in TM1 and ISP2l media when glass beads were added to favor dispersed growth (Figure S9). Indeed, adding glass beads to E26 medium cultures allowed us to use it for a successful vegetative preculture step (Figure S10A). Interestingly, routine analysis of glucose consumption in all media described above indicated that *N. coxensis* did not visibly consume glucose during growth (data not shown). We thus tested the glucose-lacking E26 (named E27), TM1 (TM1m), and ISP2l (ISP2lm) media variants for *N. coxensis* growth and putative GPA production. We found that biomass accumulation was similar in E26 and E27 (Figure S10A) and that biomass and antimicrobial production were equivalent in TM1 and ISP2l as well as in their glucose lacking variants TM1m and ISP2lm (Figure S10B and C). Currently, it is impossible to say why *N. coxensis* fails to use glucose throughout cultivation given that all necessary genes are present within its genome (Figure S10D). Thus, for all the following work with *N. coxensis*, E27, TM1m, and ISP2lm were used.

### 2.5 Expression of VanY-like Activity in *N. coxensis*

As already mentioned, the *noc* BGC encodes a Dbv7 orthologue—Noc27. We therefore tested whether d,d-carboxypeptidase activity could be detected in GPA-producing cultures of *N. coxensis*. This was measured in membrane extracts as previously reported for *N. gerenzanensis* and its mutant strains.^22^d,d-carboxypeptidase activity was measurable in *N. coxensis* extracts, although at an inferior level than in *N. gerenzanensis* (Figure S11). This indicated that Noc27 is functional and its expression correlates with the antimicrobial producing conditions. These results corroborate the hypothesis that *noc* genes are expressed and a novel GPA active versus *B. subtilis* is produced by *N. coxensis.* As in *dbv*(40) and WAC 01424 BGCs, a *vanY* gene seems to be the only cluster-situated determinant of self-resistance in *N. coxensis*.

### 2.6 Purification and Identification of the Novel Glycopeptide Complex Produced by *N. coxensis*

**Figure 4:**
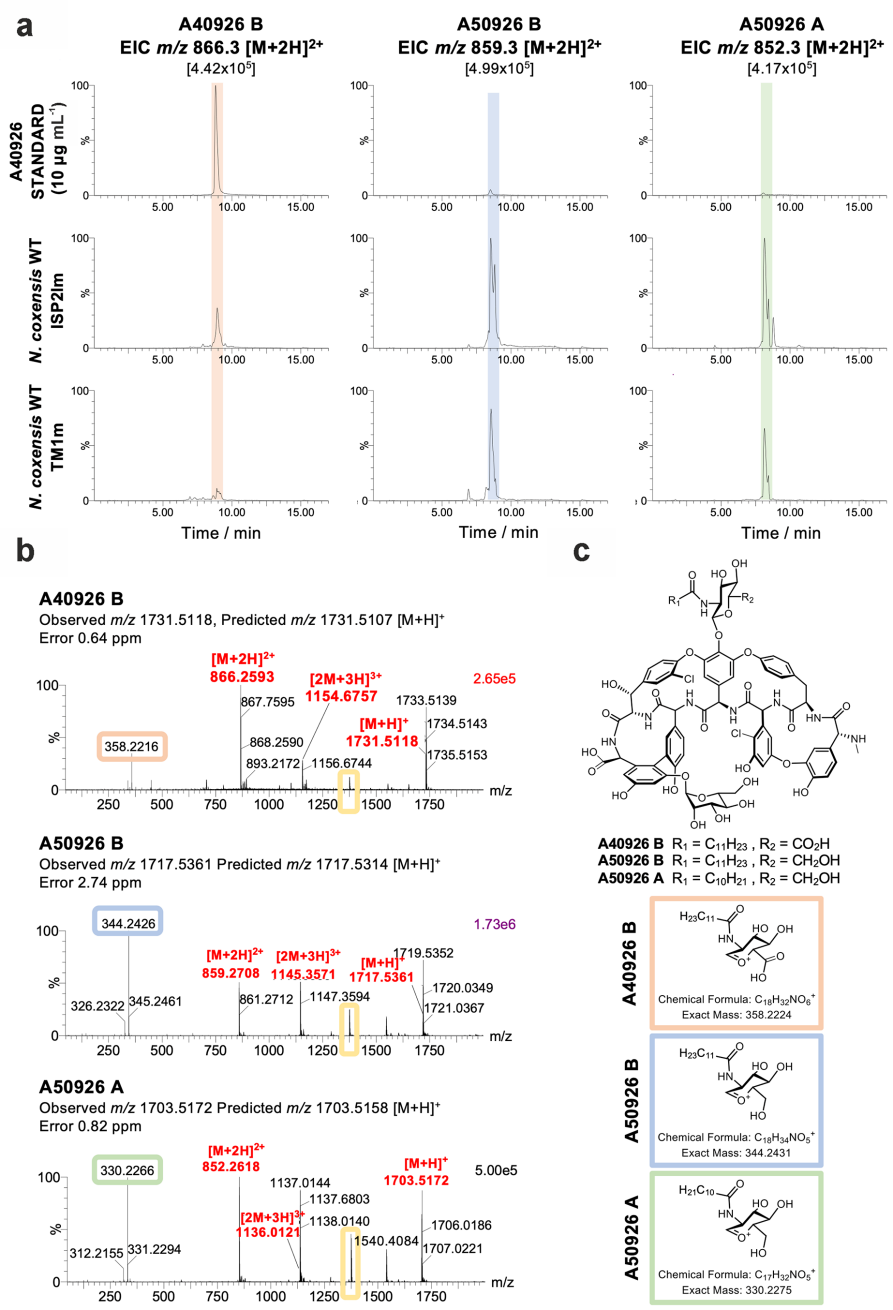
MS characterization of novel GPA complex produced by wild type *N. coxensis* grown in ISP2lm and TM1m media for 7 days. (a) Extracted ion chromatograms (EICs) of masses corresponding to A40926 B (left column) and the major components of the A50926 complex produced by *N. coxensis* WT, A50926 B (*m*/*z* 859.3, second column), and A50926 A (*m*/*z* 852.3, third column). The top row corresponds to a commercial standard of A40926 and the middle and bottom rows to culture extracts from ISP2lm and TM1m, respectively. For each mass, peak heights are normalized relative to the intensity of the largest peak in the sample set, shown in brackets at the top of each column. (b) MS spectra for A40926 B, A50926 B, and A50926 A. Peak heights are normalized to the intensity of the top peak in each spectrum, shown on the top right corner of each plot. Signature in-source fragments for each of the analyzed molecules are circled in pink, blue, and green, respectively, whereas the fragment corresponding to the mannosylated aglycone common to all of them is highlighted in yellow. (c) Proposed structure for the A50926 molecules. The top schematic represents a generic proposed structure common to A40926 and A50926 while the insets below represent the differential fragments for each of the analyzed molecules, as inferred from MS and MS/MS data.

d-Alanine-d-Alanine (d-Ala-d-Ala) affinity resin chromatography was used to capture the putative GPA from cultures of *N. coxensis* grown in ISP2lm and TM1m media. ISP2lm appeared to be the most suitable medium for GPA purification, since the rich composition and high viscosity of TM1m interfered with affinity chromatography. Analyzed by HPLC, the affinity resin eluates contained two major peaks with the characteristic UV spectra of the commercially available A40926 standard, but with a different retention time (Figure S12). LC-MS analysis of these peaks revealed they corresponded to ions with *m*/*z* 852.3 and 859.3 ([M + 2H]^2+^), 28 and 14 Da smaller respectively than an A40926 standard ([M + 2H]^2+^ = 866.3, corresponding to A40926 B). We therefore tentatively named this new GPA complex A50926 (Figure 4a and c).

All three molecules showed similar MS spectra with single, double, and triple charge proton adducts as well as in-source fragments corresponding to the aglycone carrying the mannose moiety and the Glc*N*-Acyl moiety (Figure 4b,c). The mannosylated aglycone fragment (*m*/*z* 1374.3) was common to all three peaks (Figures 4b and S13), indicating that they share the same aglycone structure and mannose decoration. In contrast, the in-source fragment corresponding to the acylated sugar carried the signature mass difference for each molecule (Figures 4b, S14, and S15): the main A50926 peak ([M + H]^+^ = 1717.5361) had a fragment with *m*/*z* 344.2, whereas the A40926 standard had a fragment with *m*/*z* 358.22 (Figures 4b, S14, and S15). Further MS and MS/MS analyses of these fragments (Figures S14 and S16) allowed us to assign this 14 Da mass difference to the glucosamine moiety. The masses are consistent with this sugar featuring a regular 6-hydroxyl group in A50926 versus being carboxylated in A40926 (Figures 1, 3, 4c, S14, S15, and S16). This correlates with the lack of a homologue of *dbv29* in the *noc* BGC, as it encodes the enzyme responsible for the oxidation of the C-6 hydroxyl group of Glc*N*-Acyl into a carboxylic acid in A40926. The second A50926 peak ([M + H]^+^ = 1703.5172) had a further 14 Da mass difference in the Glc*N*-Acyl moiety (Figures 4b and S14), but in this case MS/MS showed this difference to be in the acyl chain (Figure S16), which is consistent with an A50926 congener with a C11 acyl chain instead of a C12 acyl chain. This is equivalent to the A and B series of congeners in the A40926 complex.^41^ Based on this analysis and accurate mass data (Figure 4b), we named the compound with [M + H]^+^ = 1717.54 A50926 B (Figure 4c) and the compound with [M + H]^+^ = 1703.52 A50926 A (Figure 4c).

### 2.7 Single Gene Expression Leads to A40926 Production in *N. coxensis*

**Figure 5:**
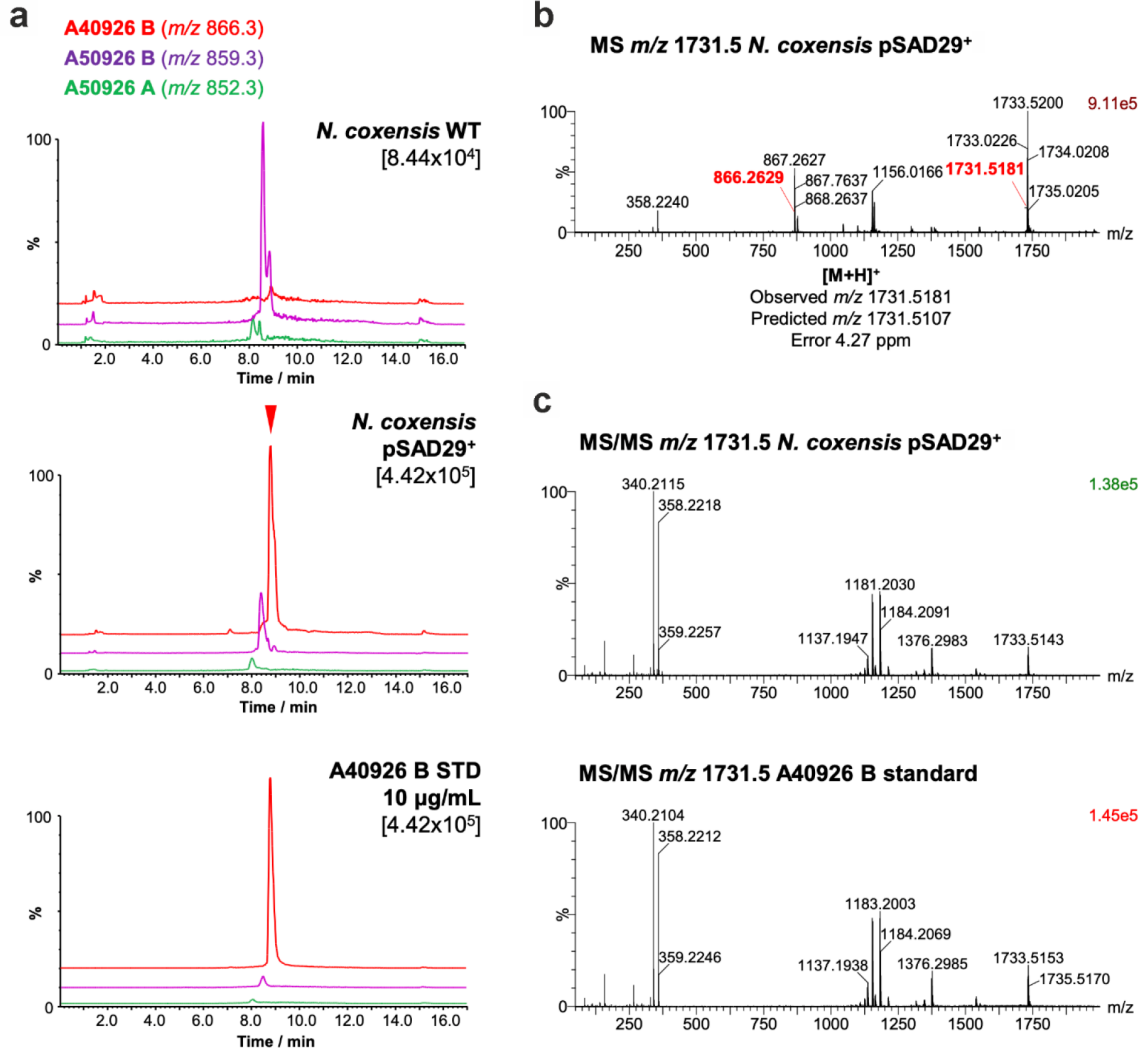
Production of A40926 in *N. coxensis* pSAD29^+^ grown in ISP2lm for 7 days. (a) EICs for masses corresponding to A40926 B (red trace), A50926 B (purple), and A50926 A (green) in purified extracts of *N. coxensis* pSAD29^+^ (top chromatogram) and *N. coxensis* WT (middle) in comparison to an A40926 commercial standard. The intensity for the top peak in each chromatogram is shown in brackets under the sample name. (b) MS spectrum of A40926 B from *N. coxensis* pSAD29^+^ cultures. Monoisotopic masses corresponding to [M + 2H]^2+^ and [M + H]^+^ adducts are highlighted in red, and the deviation between the observed accurate mass and the predicted mass for A40926 is represented in parts per million. (c) MS/MS spectra of A40926 B produced by *N. coxensis* pSAD29^+^ and an A40926 B commercial standard.

To support our MS-based characterization of A50926, we hypothesized that we could convert *N. coxensis* into an A40926 producer by overexpression of the *dbv29* gene from *N. gerenzanensis*, which encodes the hexose oxidase required for oxidation of the C-6 hydroxyl group of Glc*N*-Acyl into the corresponding carboxylic acid. To achieve this, we used the pSET152A expression platform, which has proven to be very effective for gene overexpression in both *N. coxensis* and *N. gerenzanensis*.^6^*dbv29* was cloned into pSET152A to generate pSAD29, which was then introduced into *N. coxensis* by conjugation from *Escherichia coli*. *N. coxensis* pSAD29^+^ was grown in ISP2lm medium for 168 h, and the resulting GPA complex was purified using d-Ala-d-Ala affinity resin. LC-MS analysis determined that *N. coxensis* pSAD29^+^ was able to produce a molecule with an identical retention time and MS spectrum to that of A40926 (observed *m*/*z* 1731.5181, calculated A40926 [M + H]^+^ 1731.5107, 4.27 ppm difference) (Figure 5a and b).

MS/MS analysis of the molecule showed it also had an identical fragmentation pattern to the A40926 standard, including the in-source fragment with *m*/*z* 358.22 characteristic of the carboxylated Glc*N*-Acyl moiety (Figures 5c and S17). Traces of A50926 could also be detected in the extract of the complemented strain, indicating that while complementation was very efficient, conversion from A50926 to A40926 was not complete (Figure 5a). Alongside the BGC homology (Figure 2), this provides strong evidence that A50926 is chemically identical to A40926 with the exception of the carboxylated Glc*N*-Acyl. However, we cannot completely rule out small differences, such as acyl chain branching.

### 2.8 Heterologous Expression of Transcriptional Regulators *dbv3* and *dbv4* to Enhance the Production of A50926 in *N. coxensis*

**Figure 6:**
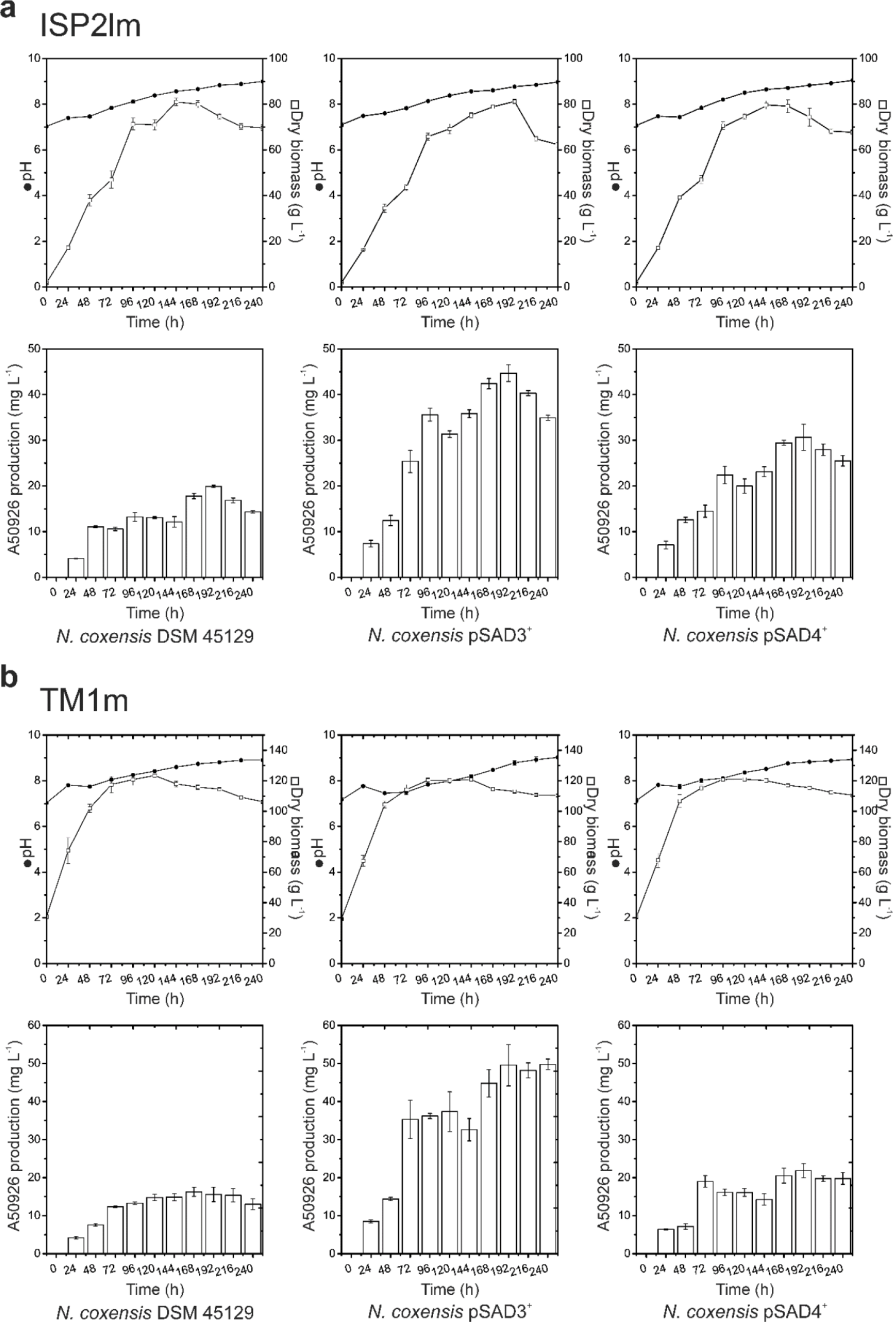
Time courses of *N. coxensis* wild type and of the recombinant strains overexpressing *dbv3* (pSAD3^+^) and *dbv4* (pSAD4^+^) cultivated in ISP2lm (a) or TM1m (b) in 500 mL Erlenmeyer flasks. pH (filled circles), biomass accumulation (empty squares), and A50926 production were monitored every 24 h. Results given are mean values of three independent experiments, and error bars represent standard deviations.

In previous work, we overexpressed the two *dbv* BGC situated master regulators in *N. gerenzanensis* (*dbv4* and *dbv3*) to successfully improve A40926 production.^6^ Therefore, hereby we used the previously constructed expression vectors pSAD4 and pSAD3 carrying *dbv4* and *dbv3*, respectively, in *N. coxensis* to trigger and improve A50926 production. First, we observed that *N. coxensis* pSAD3^+^ and pSAD4^+^ recombinant strains grown in the E27 and VSP vegetative media produced an antimicrobial activity against *B. subtilis* (Figure S18A), whereas their parental wild type strain did not exhibit any antimicrobial activity in these media. Overexpression of *dbv3* also triggered antimicrobial activity on VM0.1 and ISP2 solid media, whereas the wild type was not active (Figure S18B). Consistently, in both ISP2lm and TM1m production media *N. coxensis* pSAD3^+^ and pSAD4^+^ produced more antibiotic than the wild type (Figures S18C and 6a and b). In ISP2lm (Figure 6a), at 192 h *N. coxensis* pSAD3^+^ reached the maximum production of approximately 45 μg mL^–1^, exceeding both wild type (approximately 20 μg mL^–1^) and *N. coxensis* pSAD4^+^ (approximately 30 μg mL^–1^) productivities. In TM1m medium (Figure 6b), *N. coxensis* pSAD3^+^ produced approximately 50 μg mL^–1^ after 192 h of cultivation. At the same time point in TM1m the wild type and *N. coxensis* pSAD4^+^ produced approximately 16 and 22 μg mL^–1^ of antibiotic, respectively. The control strain carrying the “empty” pSET152A vector performed exactly as the wild type (data not shown). No significant differences between biomass accumulation or pH were observed among the recombinant strains, or in comparison with the parental *N. coxensis* wild type strain. Thus, overexpression of *dbv3* and *dbv4* regulatory genes triggered or improved the production of A50926 in *N. coxensis* under different cultivation conditions.

## 3 Conclusions

A novel GPA, A50926, was identified from *N. coxensis* DSM 45129. Detailed MS and MS/MS analysis indicates that A50926 differs from the previously characterized A40926 GPA by lacking the carboxyl group on the Glc*N*-Acyl moiety attached to Hpg4 of the GPA aglycone, resembling teicoplanin in this part of the molecule. A compound with the same chemical structure was described 25 years ago as a chemically prepared derivative of A40926 (named RA^28^). Extensive study of antibacterial activities of RA *in vitro*(28) indicated that RA has slightly better antimicrobial activity than A40926: minimal inhibitory concentrations (MICs) of RA were 2–4 times lower against different staphylococcal and enterococcal strains when compared to A40926. The difference of chemical structure between the newly described A50926 and A40926 correlates with the absence of *dbv29* orthologue in the A50926 BGC (*noc*). Consistently, when *dbv29* was introduced into *N. coxensis*, we obtained A40926 production in the recombinant strain. Otherwise, both *noc* and *dbv* BGCs share all biosynthetic genes, which are closely related. Heterologous expression of A40926 regulatory genes *dbv3* and *dbv4* in *N. coxensis* improved A50926 production.

Although the majority of *noc* and *dbv* genes are orthologous, the *dbv* BGC is significantly rearranged in comparison to the *noc* BGC, as well as all other characterized GPA BGCs. We have proposed a series of genetic inversions that could have occurred in a common *Nonomuraea* ancestor to explain these different genetic architectures. Both BGCs are quite similar to the putative GPA BGC from *Nonomuraea* sp. WAC 01424. The latter lacks genes required for the addition of mannose, but possesses a gene encoding a sulfotransferase and an additional gene encoding a halogenase. Thus, the putative nonmannosylated GPA from *Nonomuraea* sp. WAC 01424 might be sulfated and have a different chlorination pattern than A40926/A50926. Consequently, *Nonomuraea* sp. WAC 01424 GPA BGC seems an attractive source for new tailoring genes to obtain A40926 derivatives with altered pharmacological properties. Notwithstanding the GPA BGC similarity, multilocus phylogeny of *Nonomuraea* spp. shows that GPA producers are not clustered together: GPA producers are found in distinct clades within the genus. Our analysis indicates that type IV and V GPA BGCs are common in *Nonomuraea* spp., which is in contrast to how rare these BGCs were believed to be. This is comparable to studies that show that BGCs for types I–III–IV GPAs are common in *Amycolatopsis*, and type V GPAs in *Streptomyces.*(27,42,43) This highlights how rare actinomycete genera, such as *Nonomuraea*, may represent a rich untapped source of novel GPAs, as well as GPA tailoring enzymes for the diversification of existing GPA scaffolds.

## 4 Methods

### 4.1 Bacterial Strains and Cultivation Conditions

Bacterial strains and plasmids used in this work are summarized in Table S6. Compositions of all the media used for cultivation and GPA production are also given in the Supporting Information. All media components and antibiotics were supplied by Sigma-Aldrich, unless otherwise stated. For routine maintenance, *N. gerenzanensis* and *N. coxensis* strains were cultivated on ISP3 agar medium supplemented with 50 μg mL^–1^ apramycin-sulfate when appropriate. For genomic DNA isolation, *N. gerenzanensis* and *N. coxensis* strains were cultivated in liquid VSP medium on an orbital shaker at 220 rpm and at 30 °C. The working cell banks (WCBs) for *N. gerenzanensis* and *N. coxensis* strains were prepared as described previously.^22,37^*E. coli* DH5α was used as a routine cloning host, and *E. coli* ET12567 pUZ8002 was used as a donor for intergeneric conjugations. *E. coli* strains were cultivated at 37 °C in LB liquid or agar media supplemented with 100 μg mL^–1^ of apramycin-sulfate, 50 μg mL^–1^ of kanamycin-sulfate, and 25 μg mL^–1^ of chloramphenicol when appropriate.

### 4.2 Plasmid Construction and Generation of Recombinant *N. coxensis* Strains

To construct the pSAD29 expression vector, the coding sequence of *dbv29* (1601 bp) was amplified from the genomic DNA of *N. gerenzanensis* using dbv29_F/R primer pair (Table S7) and Q5 high-fidelity DNA polymerase (New England Biolabs). The resulting amplicon was digested with *Eco*RI and *Eco*RV restriction endonucleases and cloned into pSET152A^44,45^ cleaved at the same binding sites. The resulting plasmid was verified by endonuclease restriction mapping and sequencing at BMR Genomics.

pSAD29, as well as pSAD3,^6^ pSAD4,^6^ and pSET152A,^44^ were transferred to *N. coxensis* conjugatively, as described previously.^6^ Transconjugants were selected as resistant to 50 μg mL^–1^ of apramycin-sulfate. Obtained strains were verified by PCR. To verify the integration of pSAD29, a ∼1.1 kbp fragment of pSAD29 was amplified using the dbv29_seq_int/PAM_seq_R (Table S7) primer pair, in which dbv29_seq_int anneals within *dbv29* and PAM_seq_R anneals upstream the *Eco*RV cleavage site of pSET152A. To verify the integrations of pSAD4 and pSAD3, ∼1 kbp and ∼2 kbp fragments were amplified respectively using PAM_seq_F/dbv4_R and PAM_seq_F/dbv3_seq_R primer pairs (Table S7). Finally, the integration of pSET152A was verified by amplifying *aac(3)IV* with the aac(3)IV_F/R primer pair (Table S7). In all cases, genomic DNA was isolated using the Kirby procedure.^46^

### 4.3 *N. coxensis* Cultivation for A50926 Production

To initiate the cultivation of *N. coxensis*, one WCB vial was inoculated into a 250 mL Erlenmeyer flask with 50 mL of VSP reactivation medium containing 6 glass beads (ø5 mm). After 72 h of incubation on a rotary shaker at 220 rpm, 30 °C the culture was used to inoculate (10% v/v) 500 mL Erlenmeyer flasks containing 100 mL of E27 vegetative medium and 12 glass beads (⌀ 5 mm). Following 72 h of incubation on a rotary shaker at 220 rpm, 30 °C this culture was used to inoculate (10% v/v) 500 mL Erlenmeyer flasks with 100 mL of ISP2lm or TM1m production media containing 12 glass beads (⌀ 5 mm). A50926 production cultures were then incubated up to 240 h on a rotary shaker at 220 rpm, 30 °C. Samples were collected at regular time points to estimate biomass accumulation (dry weight), pH, and A50926 production.

### 4.4 VanY-Related Activity Measurement

d,d-carboxypeptidase activity in *Nonomuraea* spp. was measured in FM2 production medium (*N. gerenzanensis*) and ISP2lm (*N. coxensis*) at 24, 48, 72, 96, 120, and 144 h time points. Mycelial lysates were prepared as described previously.^34^ The enzyme activity releasing d-Ala from the tripeptide *N*-Acetyl-l-Lys-d-Ala-d-Ala (10 mM) was followed spectrophotometrically by a d-amino acid oxidase/peroxidase coupled reaction that oxidizes the colorimetric substrate 4-aminoantipyrine to chinonemine. d,d-carboxypeptidase activity was normalized to dry biomass weight, as previously reported.^22^ One unit is defined as the amount of enzyme that is able to convert 1 μmol of substrate in 1 min.

### 4.5 HPLC and LC-MS Analysis of GPAs

For quantitative measurement, A40926 and A50926 were extracted from *N. coxensis* cultures with equal volumes of borate buffer composed of 100 mM H_3_BO_3_ (Sigma-Aldrich) and 100 mM NaOH (Sigma-Aldrich), pH 12. During this extraction the *O-*acetylated forms were converted in the corresponding deacetylated GPAs A40926 and A50926. A40926 and A50926 were analyzed using HPLC as previously reported.^6,22,37^ In all cases the injection volumes of studied samples and standards were the same (50 μL). Concentration of A50926 was estimated as follows:
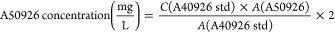
Where, *C*(A40926 std) is the concentration of the commercial A40926 sample; *A*(A50926) is the area sum of the peaks corresponding to A50926 B; *A*(A40926 std) is the area of the peak corresponding to the standard A40926 factor B_0_; and 2 is the dilution factor.

High resolution liquid chromatography–mass spectrometry (LC-MS) and fragmentation (MS/MS) analysis of A40926 and A50926 was carried out on a SYNAPT G2-Si mass spectrometer equipped with an Acquity UPLC (Waters). Samples were injected onto a Waters Acquity UPLC BEH 1.7 μm, 1 × 100 mm C18 column, and eluted with a gradient of (B) acetonitrile/0.1% formic acid in (A) water/0.1% formic acid with a flow rate of 0.08 mL min^–1^ at 45 °C. The concentration of B was kept at 1% for 2 min followed by a gradient up to 40% B over 9 min, ramping to 99% B in 1 min, kept at 99% B for 2 min and re-equilibrated at 1% B for 4 min. MS data were collected in positive mode with the following parameters: resolution mode, scan time 0.5 s, mass range *m*/*z* 50–2000 calibrated with sodium iodide, capillary voltage = 2.5 kV; cone voltage = 40 V; source temperature = 120 °C; desolvation temperature = 350 °C. Leu-enkephalin peptide was used to generate a lock-mass calibration with *m*/*z* 556.2766 for positive mode, measured every 90 s during the run. For MS/MS fragmentation, a data directed analysis (DDA) method was used with the following parameters: precursor selected from the 4 most intense ions; MS/MS threshold 5000; scan time 2 s; no dynamic exclusion. Collision energy (CE) was ramped between 8 and 35 at low mass (*m*/*z* 50) and 10–70 at high mass (*m*/*z* 1200).

### 4.6 Purification of GPAs Using d-Ala-d-Ala Based Affinity Resin

GPAs were purified by affinity chromatography with a d-Alanine-d-Alanine (d-Ala-d-Ala) based resin. Activation of 5 mL HiTrap NHS-activated HP affinity columns (GE Healthcare) and ligand binding was conducted as described before^47^ with modifications. Briefly, the resin was activated with 30 mL of 1 mM HCl, followed by injection of 200 mM d-Ala-d-Ala dipeptide, dissolved into 5 mL of coupling buffer (0.2 M NaHCO_3_, pH 7.0). After 30 min incubation, the resin was washed with three cycles of 0.5 M ethanolamine hydrochloride, 0.5 M NaCl (pH 4.0, 30 mL), followed by 0.1 M sodium acetate, 0.5 mM NaCl (pH 4.0, 30 mL), alternately. Finally, the resin was washed with 50 mL coupling buffer and left to equilibrate for at least 1 h before use.

*N. coxensis* cultures were extracted in borate buffer as reported above, the pH in the obtained extracts was adjusted to 7.5 with HCl, and they were applied to the affinity chromatography system. Thus, extracts in borate buffer, coming from *N. coxensis* strains cultivated in TM1m or ISP2lm media, were filtered with 0.45 μm cutoff and loaded onto a d-Ala-d-Ala column at a flow rate of 0.5 mL min^–1^. After extensive washing with coupling buffer, the bound GPA was eluted with 0.1 M NaOH and the eluate was lyophilized.

### 4.7 Bioassays for the Detection of A50926

Agar plug or Whatman paper disc (GE Healthcare) antibiotic diffusion assays were used to determine antimicrobial activities. An overnight *B. subtilis* ATCC 6633 culture in Mueller-Hinton broth II (cation adjusted, Sigma-Aldrich) was used to inoculate (1% v/v) a fresh culture, which was grown to OD_600_ = 0.6. A 200 μL portion of this culture was then added to 25 mL of 0.7% (w/v ) Mueller-Hinton agar (Condalab) and plated. After solidification of the media, agar plugs cut from the plates with *N. coxensis* lawns, or Whatman paper discs containing GPAs, were placed on the agar surface. Bioassay plates were incubated for 16 h at 37 °C before examination.

### 4.8 Sequencing and Annotation of the *N. coxensis* Genome

The genome of *N. coxensis* was sequenced using a combination of HiSeq Illumina and GridION ONT technologies. The Illumina data was obtained from SRA (PRJNA165411), while for the ONT data, a sequencing library (SQK-LSK109) was prepared using the Ligation Sequencing Kit (Oxford Nanopore Technologies) according to the manufacturer’s instructions and run on a GridION sequencer in an R9.4.1 flowcell (both Oxford Nanopore Technologies). Base-calling of the raw data was performed with GUPPY-FOR-GRIDION v3.0.6. The assembly and polishing were performed as described previously,^48^ using canu v.1.8 instead of v.1.6. The ONT data was assembled into 5 contigs, while the Illumina data were assembled into 87 scaffolds containing 310 contigs using NEWBLER v2.8. After manual curation using CONSED,^49^ the complete genome of *N. coxensis* DSM 45129, consisting of one circular chromosome of 9,073,954 bp (72.12% G + C) was obtained. Annotation was performed using PROKKA v1.11^50^ resulting in the prediction of 8,398 coding sequences (CDS), 5 rRNA operons, 73 tRNAs, and 5 noncoding RNA elements. The annotated genome and ONT raw data were deposited at DDBJ/ENA/GenBank under the BioProject accession number PRJNA693185.

### 4.9 *In Silico* Analysis Tools and Approaches

Routine analysis of nucleotide and amino acid sequences was performed in GENEIOUS v4.8.5.^51^ Multiple sequence alignments, selection of the best models for the phylogenetic reconstruction and phylogenetic reconstruction itself were done with the MEGA X package.^52^ To reconstruct the multilocus phylogeny of *Nonomuraea*, orthologues of 30 *S. coelicolor* house-keeping proteins (Table S3,^53^) were identified within the genomes of 34 *Nonomuraea* spp. (Table S2) using reciprocal best hit (RBH) BLAST. Sequences of these proteins from each *Nonomuraea* spp. were concatenated, and these concatenates were used for the upstream phylogenetic reconstruction.
