# Supplementary material for: Genomic-Led Discovery of a Novel Glycopeptide Antibiotic by Nonomuraea coxensis DSM 45129
Source: ACS Chem Biol. 2021 Apr 29;16(5):915–28. doi: 10.1021/acschembio.1c00170 (PMC8291499; doi:10.1021/acschembio.1c00170)
Supplement: Supplementary file 1 — cb1c00170_si_001.pdf [file cb1c00170_si_001.pdf]

Supporting Information

Genomic-led discovery of a novel glycopeptide antibiotic by *Nonomuraea coxensis* DSM 45129

Oleksandr Yushchuk[§], Natalia M.Vior[†], Andres Andreo-Vidal[§], Francesca Berini[§], Kristian Rückert[‡], Tobias Busche[‡], Elisa Binda[§], Jörn Kalinowski[‡], Andrew W. Truman[†*] and Flavia Marinelli[§*]

[§]Department of Biotechnology and Life Sciences, University of Insubria, via J. H. Dunant 3, 21100 Varese, Italy.

[†]Department of Molecular Microbiology, John Innes Centre, Norwich, NR4 7UH, United Kingdom

[‡]Technology Platform Genomics, CeBiTec, Bielefeld University, Sequenz 1, 33615 Bielefeld, Germany

* Corresponding author for chemistry Andrew W. Truman (andrew.truman@jic.ac.uk) and for microbiology Flavia Marinelli (flavia.marinelli@uninsubria.it).

Inventory of Supporting Information

Contents	Page
**Composition of media used in the study**	**3**
**Table S1.** Summary of antiSMASH analysis results for *N. coxensis* genome	**6**
**Table S2.** *Nonomuraea* spp. genomes, available in GenBank	**8**
**Table S3.** House-keeping genes used to reconstruct *Nonomuraea* MLP	**10**
**Table S4.** NRPS A-domain specificities in *dbv, noc* and related NRPS	**11**
**Table S5.** Sulphotransferases and halogenases from *dbv*, *noc* and related BGCs	**12**
**Table S6.** Bacterial strains and plasmids used in this work	**14**
**Table S7.** Oligonucleotide primers used in this work	**15**
**Figure S1.** *kis*-like GPA BGC from *Nonomuraea* sp. NN258	**16**
**Figure S2**. MLP of genus *Nonomuraea*	**17**
**Figure** S3. Comparison of the genomes of different GPA-producing *Nonomuraea* spp.	**18**
**Figure S4.** A-domain specificities of Type III-IV GPA NRPSs from *Nonomuraea* spp.	**19**
**Figure S5.** Identification of Dbv4 binding sites in *noc* BGC	**20**
**Figure S6.** Putative recombination events that led from *noc*-like BGC organization to *dbv*-like	**21**
**Figure S7.** Phylogeny of halogenases and sulphotransferases encoded within *noc, dbv* and related GPA BGCs	**22**
**Figure S8.** Putative biosynthetic pathway for a GPA from *Nonomuraea* sp. WAC 01424	**23**
**Figure S9.** Screen for GPA production conditions in *N. coxensis*	**24**
**Figure S10.** Testing different media for *N. coxensis*	**25**
**Figure S11.** VanY-related activity in *N. coxensis*	**26**
**Figure S12.** HPLC chromatograms of A50926 production in *N. coxensis*	**27**
**Figure S13.** MS analysis of the mannosylated aglycones of the A40926 and A50926	**28**
**Figure S14.** MS analysis of the GlcN-Acyl moieties of the A40926 and A50926	**29**
**Figure S15.** MS/MS analysis of A40926 B, A50926 B and A50926 A	**30**
**Figure S16.** MS/MS analysis of the GlcN-Acyl moieties of the A40926 and A50926	**31**
**Figure S17.** MS/MS analysis of A40926 commercial standard and A40926 produced in *N. coxensis* pSAD29^{+}	**32**
**Figure S18.** Bioassays showing A50926 production triggered and improved in *N. coxensis* pSAD3^{+} and pSAD4^{+}	**33**
**Supplementary references**	**34**

Composition of media used in the study:

Unless otherwise stated, components are from Sigma-Aldrich, St. Louis, MO, United States.

ISP2 (g/L of distilled water):
Yeast extract – 4;
Malt extract – 10;
Dextrose – 4;
Agar – 20;
pH 7.5.

ISP2l (g/L of distilled water):
Yeast extract – 4;
Malt extract – 10;
Dextrose – 4;
pH 7.5.

ISP2lm (g/L of distilled water):
Yeast extract – 4;
Malt extract – 10;
pH 7.5.

ISP3 (g/L of tap water):
Fine ground whole oats (Kozub, Poltava, Ukraine) – 34;
Agar – 20;
pH 7.5.

VSP (g/L of distilled water):
Soluble starch (Difco, Franklin Lakes, NJ, United States) – 24;
Dextrose – 1;
Meat extract – 3;
Yeast extract – 5;
Tryptose – 5;
L-proline – 0.5;
Sucrose – 50;
pH 7.5.

VM0.1 (g/L of distilled water):
Soluble starch (Difco) – 2.4;
Dextrose – 0.1;
Meat extract – 0.3;
Yeast extract – 0.5;
Tryptose – 0.5;
Agar – 20;
pH 7.2.

E25 (g/L of distilled water):
Dextrose – 25;

Meat extract – 4;
Yeast extract – 1;
Soybean flour – 10;
Bacto peptone – 4;
NaCl – 2.5;
pH 7.5

E26 (g/L of distilled water):
Dextrose – 25;
Soy flour – 20;
Yeast extract – 4;
NaCl – 1.25;
$CaCO_3$ – 5;
pH 7.5.

E27 (g/L of distilled water):
Soy flour – 20;
Yeast extract – 4;
NaCl – 1.25;
$CaCO_3$ – 5;
pH 7.5.

R5 (g/L of distilled water):
Sucrose – 103;
Glucose – 10;
K_2SO_4 – 0.25;
$MgCl_2$ x 6 H_2O 10.12;
Casamino acids – 0.1;
Yeast extract – 5;
TES buffer – 5.73;
Trace elements solution[1] – 2 mL
pH 7.2
ddH_2O add up to 900 ml.
To be added at the time of use:
$CaCl_2$ 3.68% (w/v) 80 mL
KH_2PO_4 0.54% (w/v) 10 mL
L-proline 20% (w/v) 15 mL

FM2 (g/L of distilled water):
Dextrose – 30;
Soy flour – 30;
Yeast extract – 8;
Malt extract – 15;
$CaCO_3$ – 5;
L-valine – 1;
pH 7.5.

TM1 (g/L of distilled water):

Dextrose – 10;
Malt extract – 30;
Yeast extract – 2.5;
Soybean flour – 15;
$CaCO_3$ – 4;
pH 7.5.

TM1m (g/L of distilled water):
Malt extract – 30;
Yeast extract – 2.5;
Soybean flour – 15;
$CaCO_3$ – 4;
pH 7.5.

Table S1. Summary of the putative BGCs predicted within *N. coxensis* genome using antiSMASH

BGC-like region	Location, bp	Most similar BGC in MIBIG database/ similarity (%)	Comments
1	219,542-229,750	desferrioxamine E BGC from *Streptomyces* sp. ID38640/100	Identical to desferrioxamine E BGC
2	309,384-330,034	blasticidin S biosynthetic gene cluster from *Streptomyces griseochromogenes*/7	Only two genes show similarity to blasticidin BGC
3	1,918,176-1,932,871	no	Putative HIV-1 Rev response element containing BGC
4	2,580,016-2,596,430	isorenieratene BGC from *Streptomyces griseus* subsp. *griseus* NBRC 13350/28	Only two genes show similarity to isorenieratene BGC
5	2,605,255-2,664,589	natamycin BGC from *Streptomyces gilvosporeus*/9	Encodes for an NRPS-PKS hybrid, two genes for ABC transporters share similarity to transporter genes from natamycin BGC
6	2,667,388-2,731,534	lysolipin I biosynthetic gene cluster from *Streptomyces tendae*/43	Contains type I PKS and NRPS genes, putative tailoring genes share similarity to lysolipin I BGC tailoring genes
7	2,813,563-2,896,967	A40926 BGC from *N. gerenzanensis*/61	*noc* BGC
8	2,931,058-2,989,541	acarviostatin I03 BGC from *Streptomyces coelicoflavus* ZG0656/22	Only six genes show similarity to acarviostatin I03 BGC
9	4,126,909-4,149,389	no	Putative class IV lanthipeptide BGC
10	3,075,747-3,094,693	chlortetracycline BGC from *Kitasatospora aureofaciens*/5	Only two genes show similarity to chlortetracycline BGC
11	4,241,436-4,337,989	kirromycin BGC from *Streptomyces collinus* Tü 365/10	Four genes show similarity to kirromycin BGC
12	5,036,130-5,090,294	meridamycin BGC from *Streptomyces* sp. NRRL 30748/5	Transporter gene shows similarity to meridamycin BGC transporter
13	5,369,623-5,479,734	butyrolactol A biosynthetic gene cluster from *Streptomyces* sp. NBRC 110030/53	Superficial similarity to butyrolactol A BGC
14	5,619,580-5,685,245	calicheamicin BGC from *Micromonospora echinospora*/2	Only two genes show similarity to calicheamicin BGC

15	6,052,873-6,094,253	mirubactin BGC from *Actinosynnema mirum* DSM 43827/21	Three transport-related genes show similarity to mirubactin BGC
16	6,544,775-6,564,867	no	Putative terpene BGC
17	6,756,057-6,797,112	lagunapyrone A BGC from *Streptomyces* sp. MP131-18/22	Only two genes show similarity to lagunapyrone BGC
18	7,117,439-7,169,843	no	Putative NRPS/PKS BGC
19	7,766,570-7,773,806	no	Putative siderophore BGC
20	7,947,755-7,958,612	no	Putative RiPP BGC
21	7,975,769-7,998,173	no	Putative lassopeptide BGC
22	8,119,609-8,148,927	no	Putative betalctone BGC
23	8,163,803-8,209,012	chlorothricin BGC from *Streptomyces antibioticus*/6	Only two genes show similarity to chlorothricin BGC
24	8,274,638-8,320,463	murayaquinone BGC from *Streptomyces griseoruber*/3	One gene show similarity to murayaquinone BGC
25	8,672,394-8,685,664	no	Putative siderophore BGC
26	8,703,672-8,724,947	geosmin BGC from *Nostoc punctiforme* PCC 73102/100	Identical to geosmin BGC
27	8,874,658-8,903,559	hopene BGC from *Streptomyces coelicolor* A3(2)	Six genes show similarity tp hopene BGC

Table S2. *Nonomuraea* genomes available in GenBank.

Strain	Assembly accession number	Assembly state
Nonomuraea sp. ATCC 39727 (=*N. gerenzanensis* ATCC 39727)	LT559118.1 (GenBank accession)	complete genome
Nonomuraea polychroma DSM 43925	ASM401150v1	single contig
Nonomuraea sp. FMUSA5-5	ASM1203427v1	contigs
Nonomuraea sp. 160415	ASM589312v1	contigs
Nonomuraea sp. SBT364	*Nonomuraea* sp. SBT364_SPAdes_SSPACE	scaffolds
Nonomuraea sp. KC712	ASM434901v1	contigs
Nonomuraea sp. 6K102	ASM435280v1	contigs
Nonomuraea sp. K271	ASM990821v1	contigs
Nonomuraea sp. KC310	ASM434868v1	contigs
Nonomuraea sp. KC401	ASM577475v1	contigs
Nonomuraea sp. CH32	ASM434899v1	contigs
Nonomuraea sp. KC201	ASM434834v1	contigs
Nonomuraea sp. KC333	ASM323639v1	contigs
Nonomuraea sp. NEAU-YG30	ASM331339v1	contigs
Nonomuraea kuesteri NRRL B-24325	ASM71613v1	contigs
Nonomuraea sp. PA05	ASM808604v1	contigs
Nonomuraea sp. p1410	ASM976092v1	scaffolds
Nonomuraea sp. WAC 01424	ASM394731v1	contigs
Nonomuraea sp. NBRC 110462	ASM141775v1	few scaffolds
Nonomuraea sp. C10	ASM801751v1	few contigs
Nonomuraea sp. WYY166	ASM949707v1	complete genome
Nonomuraea sp. ATCC 55076	ASM205745v1	complete genome
Nonomuraea zeae DSM 100528	ASM588972v1	contigs
Nonomuraea turkmeniaca DSM 43926	ASM588973v1	contigs
Nonomuraea indica DRQ-2	ASM285074v1	scaffolds
Nonomuraea maritima CGMCC 4.5681	IMG-taxon 2675903066 annotated assembly	scaffolds
Nonomuraea coxensis DSM 45129	ASM37988v1	scaffolds
Nonomuraea candida NRRL B-24552	Doro.v1.0	scaffolds
Nonomuraea jiangxiensis CGMCC 4.6533	IMG-taxon 2675903065 annotated assembly	scaffolds
Nonomuraea pusilla DSM 43357	IMG-taxon 2599185218 annotated assembly	scaffolds
Nonomuraea wenchangensis CGMCC 4.5598	IMG-taxon 2675903060 annotated assembly	scaffolds
Nonomuraea fuscirosea CGMCC 4.7104	ASM300193v1	contigs

Nonomuraea solani CGMCC 4.7037	IMG-taxon 2675903140 annotated assembly	contigs
Nonomuraea phyllanthi PA1-10	ASM633498v2	contigs

Table S3. Set of house-keeping proteins used for reconstruction of multi locus phylogeny (MLP) of *Nonomuraea* species and locus tags of their orthologues in *S. coelicolor*.

**Protein**	**Protein in *S. coelicolor***
30S ribosomal protein S5 201	SCO4719
Dimethyladenosine transferase (KsgA) 286	SCO3149
Ribosomal protein S13 126	SCO4727
30S ribosomal protein S7 156	SCO4660
50S ribosomal protein L13P 147	SCO4734
Elongation factor Tu 397	SCO4662
30S ribosomal protein S9 170	SCO4735
50S ribosomal protein L11 144	SCO4648
30S ribosomal protein S15 95	SCO5736
50S ribosomal protein L1 241	SCO4649
Ribosomal protein S19 93	SCO4706
Gyrase B 686 RNA polymerase β' subunit (RpoC) 1299	SCO4655
Gyrase A 857 50S	SCO3873
50S ribosomal protein L10/L16 176	SCO4652
Ribosomal protein L4 219	SCO1505
Preprotein translocase subunit SecY 437	SCO4722
Ribosomal protein L2 278	SCO5624
RNA polymerase subunit alpha 340	SCO4792
50S ribosomal protein L22 125	SCO4707
Threonyl-tRNA-synthetase 658	SCO3778
30S ribosomal protein S3 277	SCO4708
Molecular chaperone DnaK (Hsp70) 618	SCO3671
50S ribosomal protein L14 122	SCO4712
Chaperonin GroEL (Hsp60) 541	SCO4762
Ribosomal protein L5 185	SCO4714
O-sialoglycoprotein endopeptidase	SCO4752
374 30S ribosomal protein S8 132	SCO4716
Phosphatidate cytidylyltransferase 391	SCO5628
50S ribosomal protein L6 179	SCO4717
CDP-diglyceride synthase (CdsA) 391	SCO5628
Ribosomal protein L3 214	SCO4702

Table S4. Prediction of NRPS A-domain specificities in *noc*-encoded and related GPA NRPSs; Hpg – 4-hydroxyphenylglycine; Tyr – tyrosine; Dpg – 3,5-dihydroxyphenylglycine; Bht – β-hydroxytyrosine. A-domain specificities were predicted using NRPSpredictor2[2].

BGC	NRPS	Position of the A-domain	Extracted Stachelhaus code	A-domain specificity prediction, based on the Stachelhaus code
dbv	Dbv25	A1	DAFHLGLLCK	Hpg
		A2	DASTVAAVCK	Tyr
	Dbv26	A3	DAYNAGTLCK	Dpg
	Dbv17	A4	DIFHLGLLCK	Hpg
		A5	DALHLGLLCK	Hpg
		A6	DASTVAAVCK	Tyr
	Dbv16	A7	DPYHGGTLCK	Dpg
noc	NocA	A1	DAFHLGLLCK	Hpg
		A2	DASTVAAVCK	Tyr
	NocB	A3	DAYNAGTLCK	Dpg
	NocC	A4	DIFHLGLLCK	Hpg
		A5	DALHLGLLCK	Hpg
		A6	DASTVAAVCK	Tyr
	NocD	A7	DPYHGGTLCK	Dpg
WAC01424 GPA BGC	WP_125645195	A1	DAYHLGLLCK	Hpg
		A2	DASTVAAVCK	Tyr
	WP_125645193	A3	DAYNAGTLCK	Dpg
	WP_125645191	A4	DIFHLGLLCK	Hpg
		A5	DALHLGLLCK	Hpg
		A6	DASTVAAVCK	Tyr
	WP_125645189	A7	DPYHGGTLCK	Dpg
tei	TeiA	A1	DAFHLGLLCK	Hpg
		A2	DASTVAAVCK	Tyr
	TeiB	A3	DAYNLGTLCK	Dpg
	TeiC	A4	DIFHLGLLCK	Hpg
		A5	DALHLGLLCK	Hpg
		A6	DASTIAGVCK	Tyr
	TeiD	A7	DPYHGGTLCK	Dpg
NRRL 2430 ristocetin BGC	AIE77057.1			
		A1	DACHLGLLCK	Hpg
		A2	DTSKTAAICK	Bht
	AIE77058.1	A3	DPYNQGTFCK	Dpg
	AIE77059.1	A4	DIFHLGLLCK	Hpg
		A5	DAVHLGLLCK	Hpg
		A6	DASTLGAICK	Bht
	AIE77060.1	A7	DPYHGGTLCK	Dpg

Table S5. Sources and accession numbers of GPA BGC-encoded halogenases and sulphotransferases used for the phylogenetic reconstruction in Figure S7.

Sulphotransferase	Protein accession number	Source
Teg12	ACJ60995	Uncultured soil bacterium clone D30 TEG GPA biosynthetic gene cluster (BGC)
Teg13	ACJ60996	Uncultured soil bacterium clone D30 TEG GPA BGC
Teg14	ACJ60997	Uncultured soil bacterium clone D30 TEG GPA BGC
n/a	AGO98990	*Streptomyces* sp. WAC4229 pekiskomycin BGC
Pek25	AGF91760	*Streptomyces* sp. WAC1420 pekiskomycin BGC (*pek*)
n/a	AGS49779	Uncultured bacterium esnapd15 GPA BGC
StaL	AAM80529	*Streptomyces toyocaensis* NRRL 15009 A47934 BGC (*sta*)
n/a	WP_125645175	*Nonomuraea* sp. WAC01424 GPA BGC
Auk20	AGS77324	*Actinoplanes* sp. ATCC 53533 UK-68,597 BGC (*auk*)
n/a	WP_030455526	*Herbidospora cretaceae,* sulphotransferase with unknown function

Halogenase	Protein accession number	Source
Veg13	ACJ60955	Uncultured soil bacterium clone B128 VEG GPA BGC
VhaA	CCD33142	*Amycolatopsis orientalis* ATCC19795 vancomycin BGC
BhaA	CAA76550	*Amycolatopsis balhimycina* DSM 5908 balhimycin BGC
CepH	n/a	*Amycolatopsis orientalis* chloroeremomycin BGC (*cep*)
n/a	AGO98992	*Streptomyces* sp. WAC4229 pekiskomycin BGC
Pek27	AGF91762	*Streptomyces* sp. WAC1420 pekiskomycin BGC (*pek*)
	WP_125645180	*Nonomuraea* sp. WAC01424 GPA BGC
Teg16	ACJ60999	Uncultured soil bacterium clone D30 TEG GPA biosynthetic gene cluster (BGC)
Dbv10	CAD91205	*Nonomuraea gerenzanensis* ATCC 39727 A40926 BGC (*dbv*)
Noc24	n/a	*Nonomuraea coxensis* DSM 45129 A50926 BGC (*noc*)
ComH	AAK81830	*Streptomyces lavendulae* complestatin BGC (*com*)
KisU	AQZ69237	*Nonomuraea* sp. ATCC 55076 kistamicin BGC (*kis*)
n/a	WP_173522348	*Nonomuraea* sp. NN258 *kis*-like GPA BGC
Auk21	AGS77325.1	*Actinoplanes* sp. ATCC 53533 UK-68,597 BGC (*auk*)
n/a	AGS49782	Uncultured bacterium esnapd15 GPA BGC
Tei8	CAG15020.1	*Actinoplanes teichomyceticus* ATCC 31121 teicoplanin BGC (*tei*)
CA915-35	ADU56156	Uncultured organism CA915 GPA BGC
Auk23	AGS77327.1	*Actinoplanes* sp. ATCC 53533 UK-68,597 BGC (*auk*)
CA37-38	ADU56061	Uncultured organism CA37 GPA BGC
n/a	WP_125645176	*Nonomuraea* sp. WAC01424 GPA BGC
n/a	AGS49780	Uncultured bacterium esnapd15 GPA BGC
StaK	AAM80530.1	*Streptomyces toyocaensis* NRRL 15009 A47934 BGC (*sta*)

SCO1275	2SCG18	*Streptomyces coelicolor* A3(2), halogenase with unknown function

n/a – not available.

Table S6. Bacterial strains and plasmids used in this work.

Name	Description	Source or reference
N. gerenzanensis	Wild type, A40926 producer	ATCC 39727
N. coxensis	Wild type, A50926 producer	DSM 45129
B. subtilis	GPA test-culture	ATCC 6633
N. coxensis pSET152A$^+$	Wild type derivative carrying pSET152A	This work
N. coxensis pSAD3$^+$	Wild type derivative carrying pSAD3	This work
N. coxensis pSAD4$^+$	Wild type derivative carrying pSAD4	This work
N. coxensis pSAD29$^+$	Wild type derivative carrying pSAD29	This work
E. coli DH5α	General cloning host	MBI Fermentas
E. coli ET12567 (pUZ8002$^+$)	(*dam-13*::Tn9 *dcm-6*), pUZ8002$^+$ (Δ*oriT*), used for conjugative transfer of DNA into *Nonomuraea* spp.	1
pSET152A	pSET152 derivative, containing *aac(3)IVp* from pIJ773	3, 4
pSAD3	pSET152A derivative, containing *dbv3* under the control of *aac(3)IVp*	5
pSAD4	pSET152A derivative, containing *dbv4* under the control of *aac(3)IVp*	5
pSAD29	pSET152A derivative, containing *dbv29* under the control of *aac(3)IVp*	This work

Table S7. Oligonucleotide primers used in this work.

Primer	Nucleotide sequence (5’-3’)*	Purpose
dbv29_F	TTTGATATCGGAGGGCGGTGGTGACCGGCGGCAC	Cloning of *dbv29* into pSET152A
dbv29_R	TTTGAATTCTCAGGGCCGGATCGACAACGCG	
PAM_seq_F	GATGTCATCAGCGGTGGAG	Verification of recombinant strains
PAM_seq_R	TGAGCGGATAACAATTTCA	
dbv29_seq_int	TGTCACGGCAGTTCGGCTC	
dbv3_seq_R	CCAGCGCTGGACCGCCTGC	
dbv4_R	TTTGAATTCTCCACTCGTGCTCATCCAG	
aac(3)IV_F	ATCGACTGATGTCATCAGCG	Amplification of *aac(3)IV*
aac(3)IV_R	CGAGCTGAAGAAAGACAAT	

* recognition sites of restriction endonucleases are underlined.

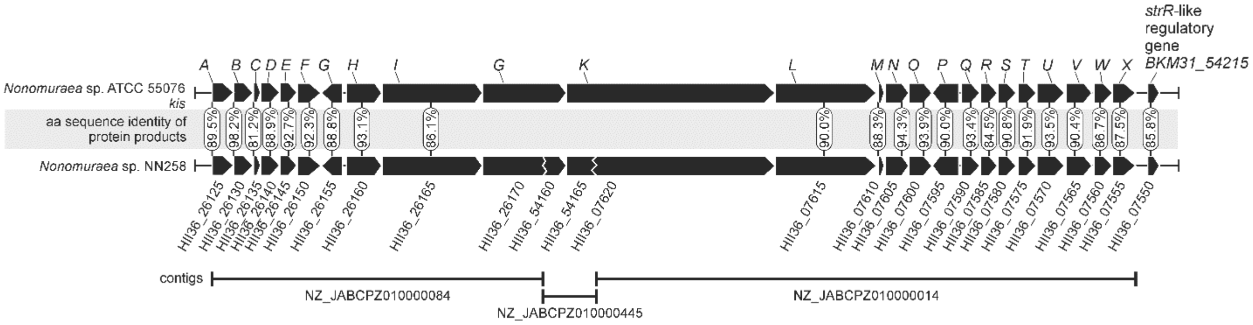

Figure S1. Comparison of kistamicin – *kis*[6] – BGC from *Nonomuraea* sp. ATCC 55076 with the putative type V GPA BGC from *Nonomuraea* sp. NN258. The sequence of the latter was reconstructed from three contigs of the *Nonomuraea* sp. NN258 genomic draft. Both clusters share an identical genetic organization and the same numbers of genes; the corresponding protein products possess high levels of amino acid sequence identity.

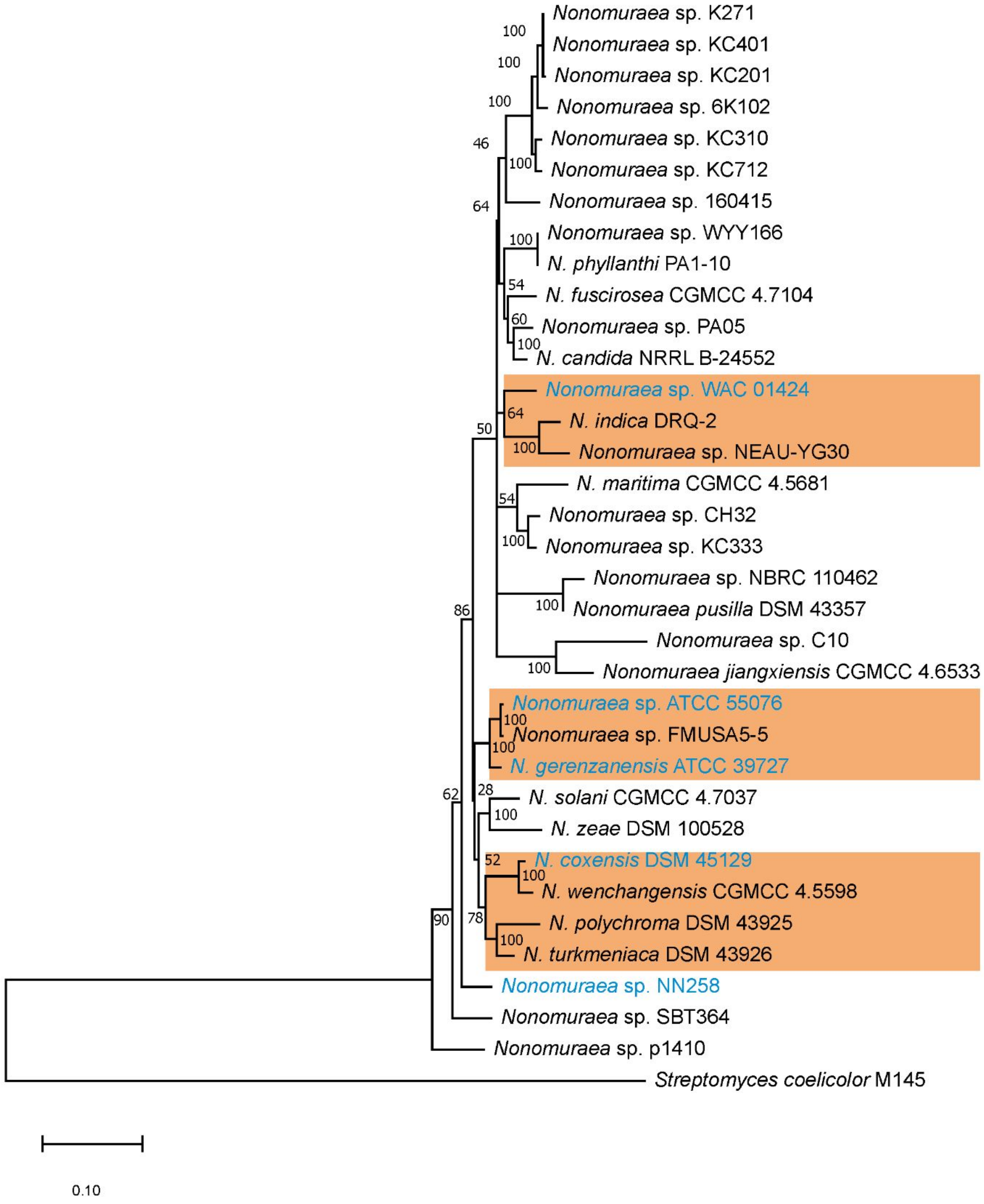

Figure S2. MLP tree of *Nonomuraea* spp. whose genomic records are available (Table S2). Well-supported clades containing GPA producers are highlighted in orange. Strains known to possess GPA BGCs in their genomes are in blue. The tree was built using concatenated protein sequences of 30 housekeeping proteins, orthologous to *S. coelicolor* proteins given in Table 3S. Phylogeny was inferred using Mega X[7] with Maximum Likelihood method and JTT matrix-based model with Gamma distribution and invariable sites; final topology and bootstrap support values (indicated at the base of the branching points) are based on 500 bootstraps.

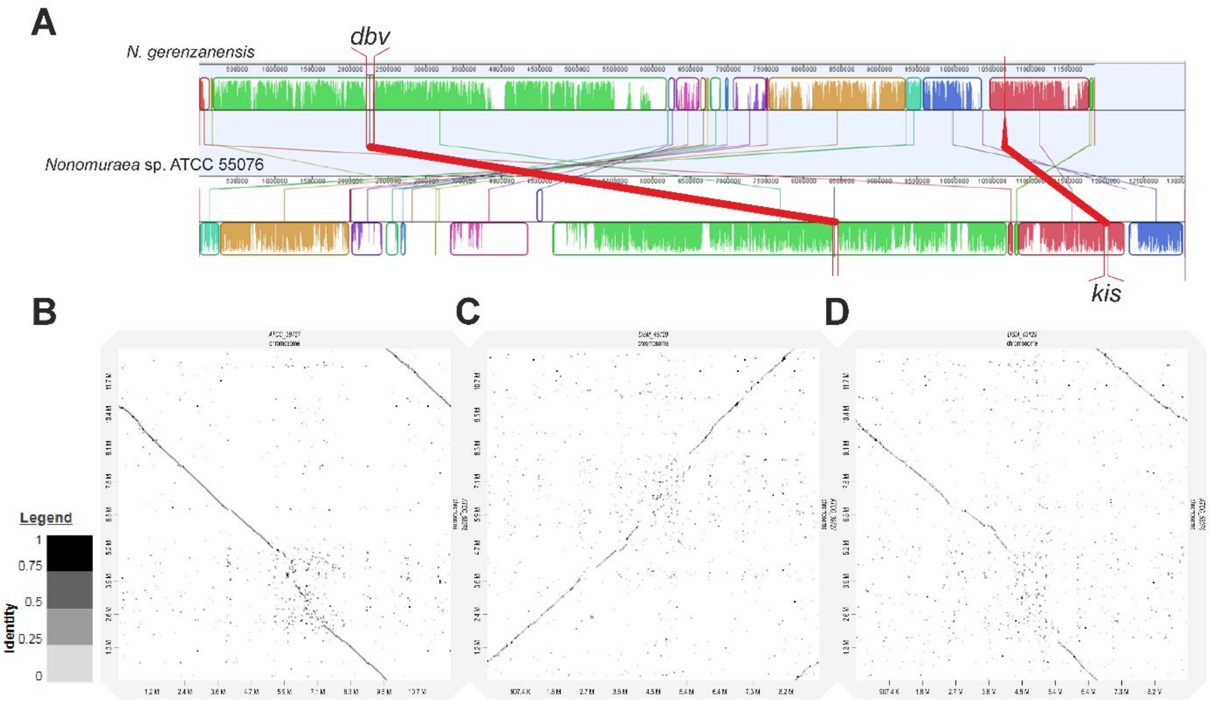

Figure S3. (A) MAUVE[8] alignment of *N. gerenzanensis* and *Nonomuraea* sp. ATCC 55076 genomes; significant similarity of both genomes could be observed. Location of the insertion loci for the *dbv* and *kis* BGCs is indicated in red. (B) Dot plots of the *N. gerenzanensis* and *Nonomuraea* sp. ATCC 55076 chromosomes showing similarity of both genomes. (C) Dot plots of *N. coxensis* and *Nonomuraea* sp. ATCC 55076 and (D) of *N. coxensis* and *N. gerenzanensis,* showing that the genome of *N. coxensis* is less similar to the other two. Dot plots were constructed using D-genies[9].

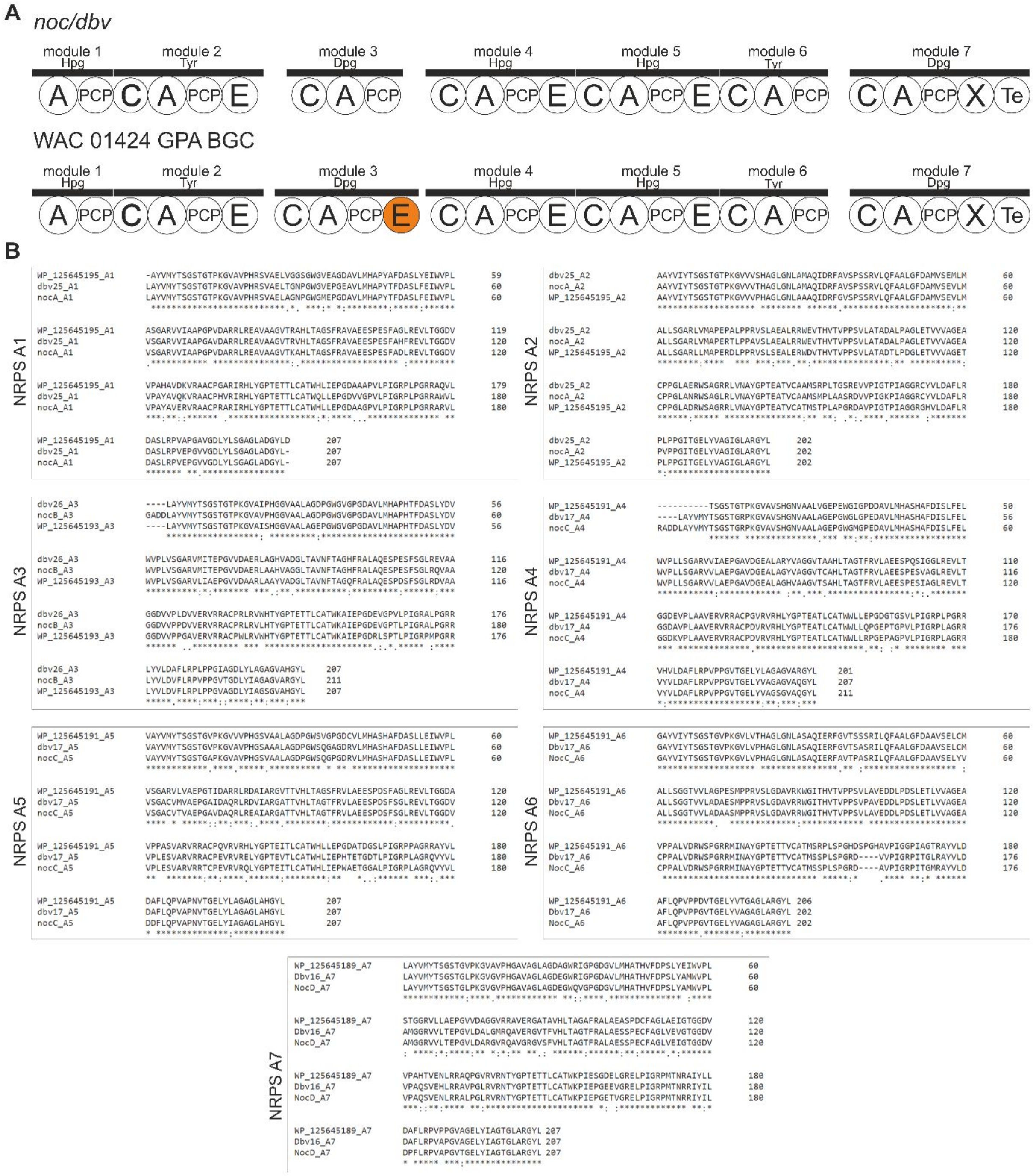

Figure S4. (A) Organization of the NRPSs encoded in *noc, dbv* and WAC 01424 GPA BGCs. Epimerization (E-) domain in module 3 of WAC 01424 GPA NRPS (orange) might be non-functional. (B) Sequence alignments of the seven A-domains of the three NRPSs, which are highly similar and share the same amino acid specificity (see also Table S4). Alignments were built in Clustal Omega[10]. Hpg – 4-hydroxyphenylglycine; Tyr – tyrosine; Dpg – 3,5-dihydroxyphenylglycine.

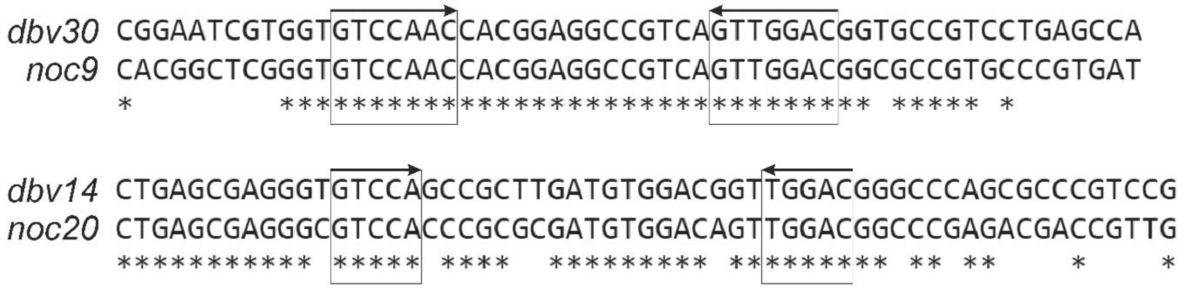

Figure S5. Pairwise alignments of the putative Dbv4 binding regions[11] found in the promoters of *dbv30* and *noc9* as well as in *dbv14* and *noc20*. Inverted repeats are in boxes with arrows, asterisks indicate identical nucleotides. Alignment was built with Clustal Omega[10].

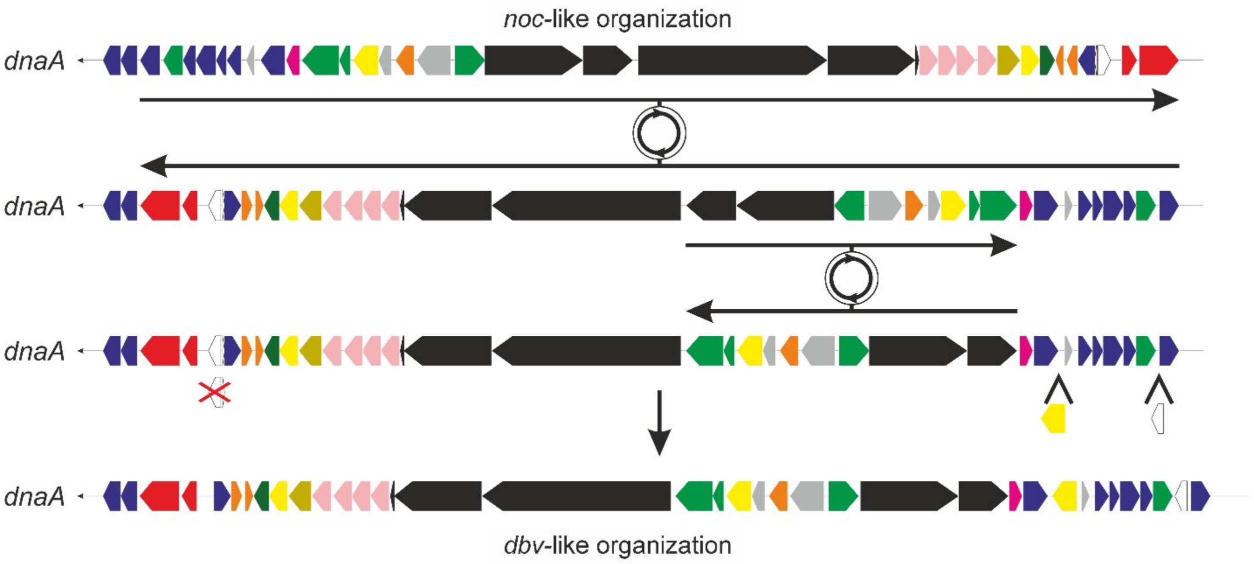

Figure S6. A scheme for the proposed recombination events involving two chromosomal inversions (as well as gene loss/gain events) that may have led to the uncommon organization of *dbv* BGC, starting from the *noc* BGC organization which is similar to those of other know GPA BGCs. In the *dbv* BGC, NRPS genes are encoded on different strands and are separated by other biosynthetic genes, which contrasts with the *noc* and other known GPA BGCs.

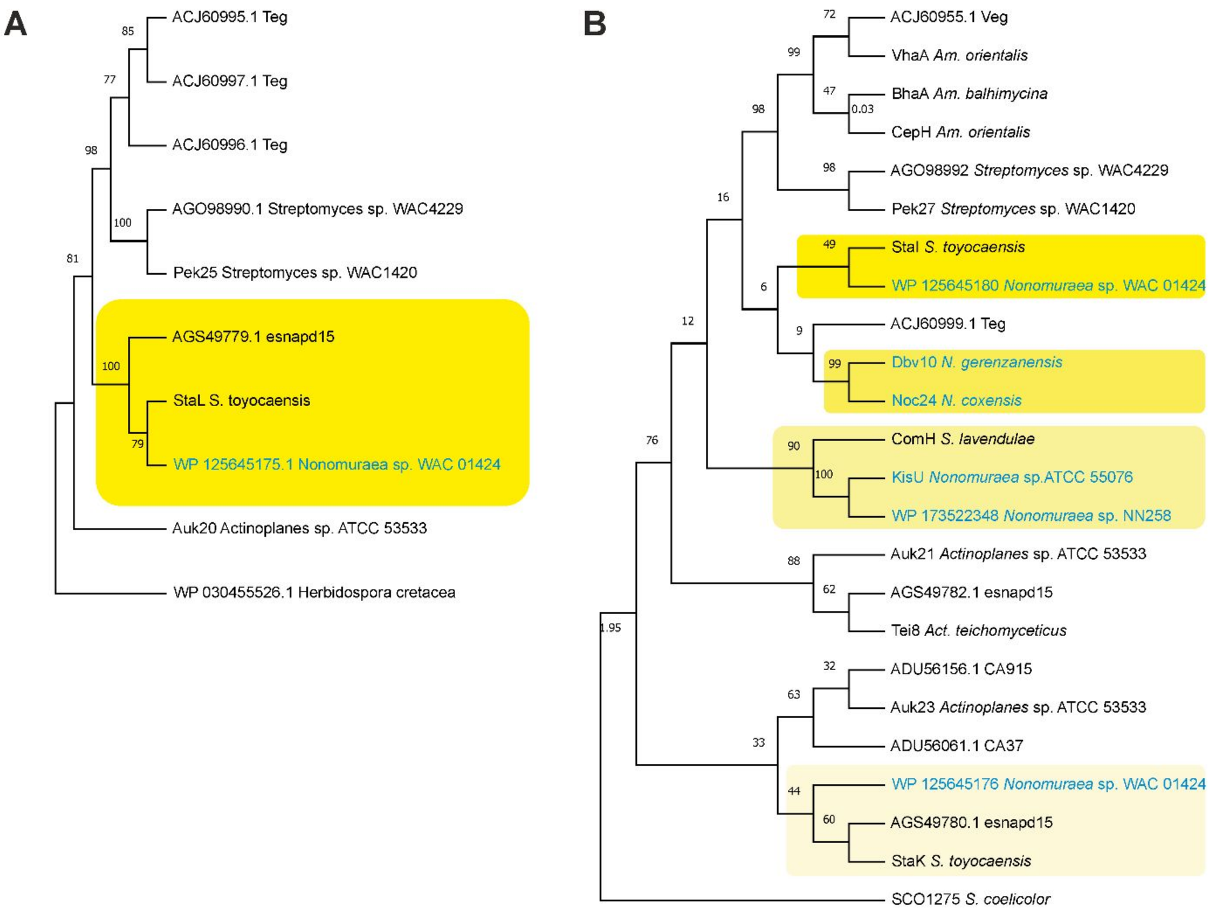

Figure S7. (A) Cladogram of the sulphotransferases, encoded within GPA BGCs. The sulphotransferase encoded within WAC 01424 putative GPA BGC (blue) is closely related to the one encoded within A47934 BGC (StaL) from *Streptomyces toyocaensis.* (B) Cladogram of the halogenases encoded within GPA BGCs. Halogenases from *Nonomuraea* spp. are in blue. Clades grouping different halogenases from *Nonomuraea* spp. are highlighted in different shades of yellow. The two halogenases encoded within *Nonomuraea* sp. WAC 01424 GPA BGC are sharing clades with halogenases coded within A47934 BGC – StaI and StaK, being distantly related to Dbv10 and Noc24. Phylogeny was inferred using Mega X[7] with Maximum Likelihood method and JTT matrix-based model; final topologies and bootstrap support values (indicated at the base of the branching points) are based on 500 bootstraps. Detailed information for the amino acid sequences used in this reconstruction is given in Table S5.

A

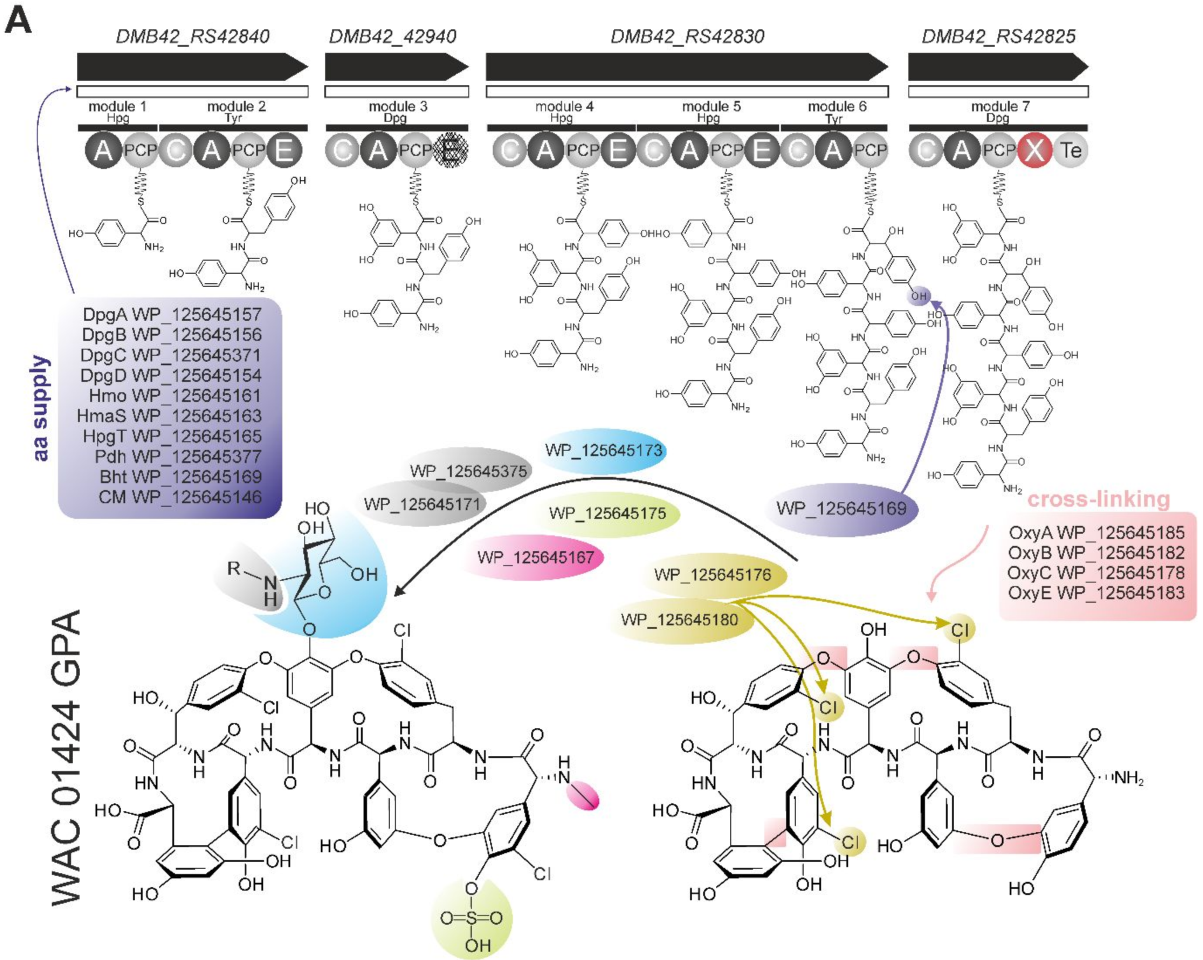

B

```
WAC       GELPWTPVMRLWGDGVASAGFAQWVVVGAPSDLTEKVLVAGVAALVDTHDMLRLRVQ---      57
A47934    GDVARTPMTKVWGPG-----AARWVTVGAPGDLSVDTLAAGLSVVIDAHDMLRSRTVGAG      55
          *::  **: ::** *      *:**.****.**: ..*.**::.::*:***** *.

WAC       SGRLVVGERGSVDAARLVRRVDG--GSLQEVV----ENAVRRLNPAAGVVAQVVWMDAGP      111
A47934    TPRMVVSGRGAVDAAHLIERVEADTGDLDDVAERCAREAAGRLDPAAGVMVRAVWVDAGP      115
          : *:**. **:****:*:.**:.  *.*::*.    .:*. **:*****:.:.**:****
                     "His-motif"
                     HHxxxDxxSW
WAC       DRVGRLVVVAHHLAVDEASWRVLVPDLRAACEGE-------PEPVGMSFRRWAGLLEEWA      164
A47934    GRVGRLVLVAHPAVVDARSWEILLRDLRAACEAVAAGREPSVDPVDVSFRRWAAALAEQA      175
          .******:***   .**  **.:*: *******.         :**.:******. * * *

WAC       VSAERAKELAAWKAIVGEDDQPIGQGLSRGKVRRRSWVVSGAEASVLAGRAPAVFFCDVQ      224
A47934    TSAERVAELTGWTALLDGEEPRLGEPGTGSPAGRRSWTLPKDRAGALAEQVTSAFHCGVH      235
          .****. **:.*.*::. ::  :*:  : . . ****.:   .*..** :. :.*.*.*:

WAC       DVLLAGLAGSVARWRGADVVLVDLEGHGRVP----DVSRTVGWFTSVHPVRLDM      274
A47934    EVLLTGLAGAVARWRGGHVVLVDVEDHGRHPVGGMDPSRTVGRFTSVHPVRLDV      289
          :***:****:******..*****:*.*** *    * ***** **********:
```

Figure S8. (A) Putative biosynthetic route encoded by WAC 01424 GPA BGC and proposed structure of the putative resulting GPA. (B) Amino acid sequence alignment of module 3 E-domain (crosshatched) from A47934 biosynthesis NRPS and WAC 01424 GPA biosynthesis NRPS. We cannot rule out that module 3 E-domain might be non-functional, like in the case of NPRS module 3 E-domain involved in A47934 biosynthesis from *Streptomyces toyocaensis* NRRL 15009[12]; however, the "His-motif" (HHxxxDxxSW, involved in the racemase activity[13]) in WAC 01424 GPA module 3 E-domain seems to be intact, differently from the A47934 NRPS module 3 E-domain.

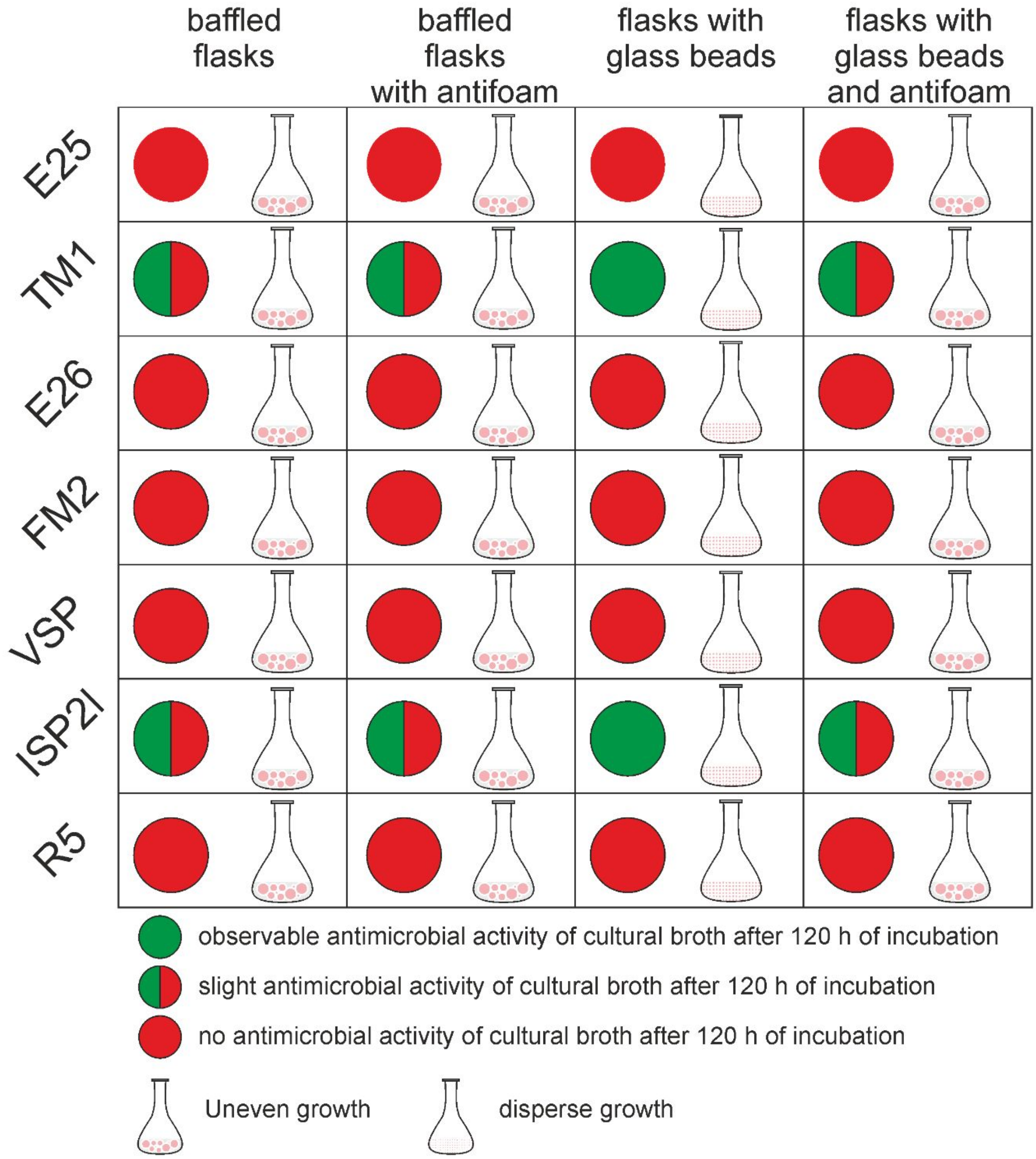

Figure S9. Scheme representing the growth pattern and the cell-free broth antimicrobial activity of *N. coxensis* cultivated in different media. Antimicrobial activity production was observed in two media (TM1 and ISP2l) out of the seven tested. In these media, antimicrobial activity seemed correlated with the mycelium dispersed growth that was observed only under cultivation in Erlenmeyer flasks with the addition of 5 mm glass beads. When baffled flasks were used and antifoam was added, culture tended to form irregular mycelial pellets and antimicrobial activity was negatively affected. Antimicrobial activity was tested against *B. subtilis* ATCC 6633 in Whatman paper disc antibiotic diffusion assays.

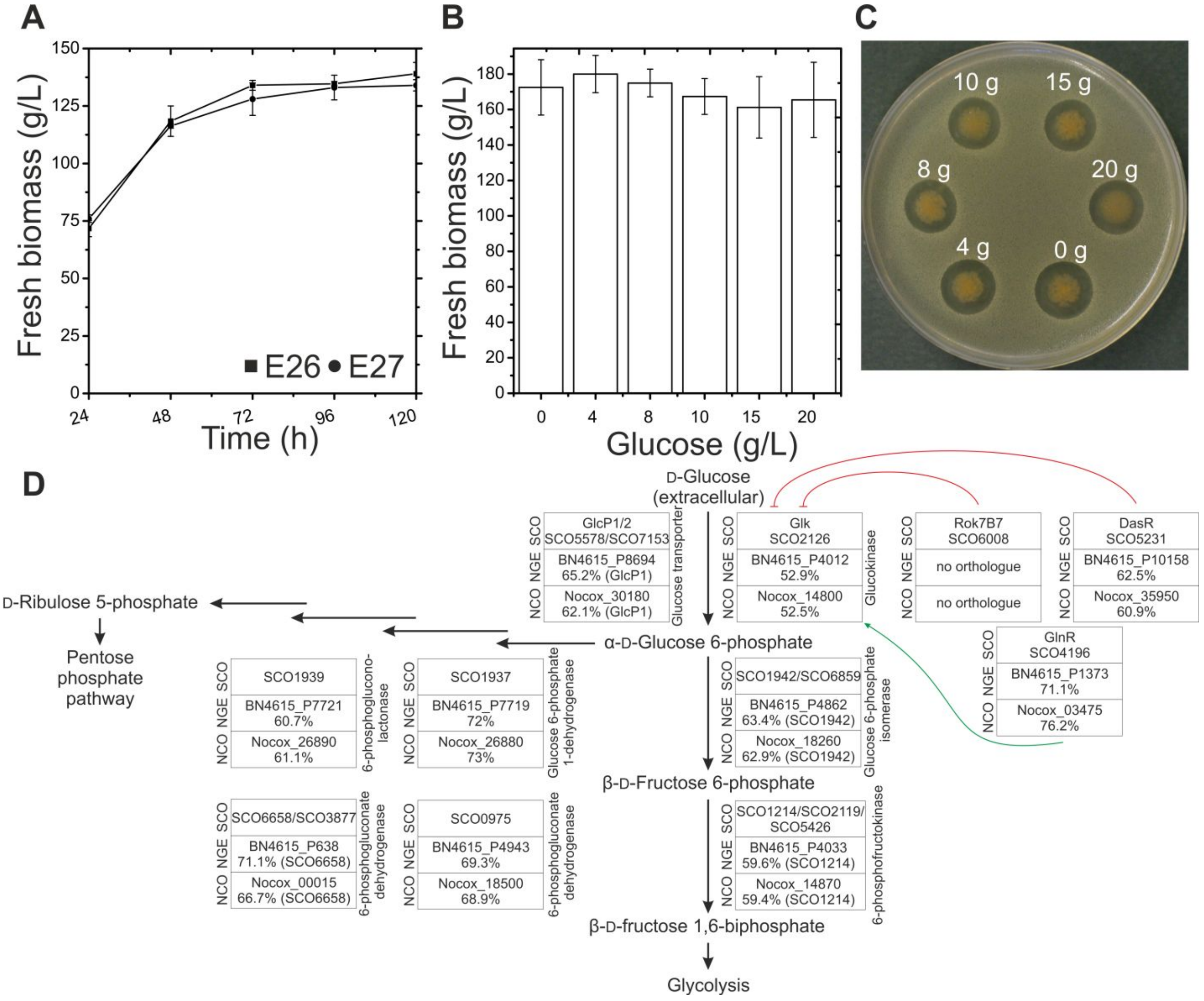

Figure S10. (A) Biomass accumulation of *N. coxensis* in E26 culture medium and its glucose-lacking variant E27. Growth curves were similar in the two vegetative media and biomass produced after 72 h of cultivation was used to inoculate production media. (B) Biomass accumulation in *N. coxensis* cultures grown for 120 h in ISP2l medium supplemented with increasing concentrations of glucose. The variant lacking glucose was named ISP2lm. (C) Antimicrobial activities of culture broths of *N. coxensis* grown in ISP2l media with different glucose concentrations (g L^{-1}) against *B. subtilis* ATCC 6633; in this bioassay 50 μL of production culture were placed on the surface of 0.7% Mueller-Hinton agar plate containing 10^6 *B. subtilis* cells. In both (B) and (C), the addition of different concentrations of glucose (from 0 to 20 g L^{-1}) to ISP2l did not exert any significant impact on either biomass accumulation or antimicrobial activity. Similar results were obtained also in TM1 and TM1m lacking-glucose (data not shown). (D) Comparative genomics of early steps of glucose metabolism in *N. coxensis* (NCO), compared to *N. gerenzanensis* (NGE) and *S. coelicolor* (SCO). All key enzymes of glucose catabolism (as well as some regulators) from *S. coelicolor* have orthologues in both *Nonomuraea* spp.; orthologues were discovered using Reciprocal Best Hit (RBH) BLAST approach, percent of amino acid sequence identity of *Nonomuraea* proteins to *S. coelicolor* proteins is shown under locus tag identifiers. Inability of *N. coxensis* to consume glucose might stem from the lack of expression of some of these genes and requires further experimental evaluation.

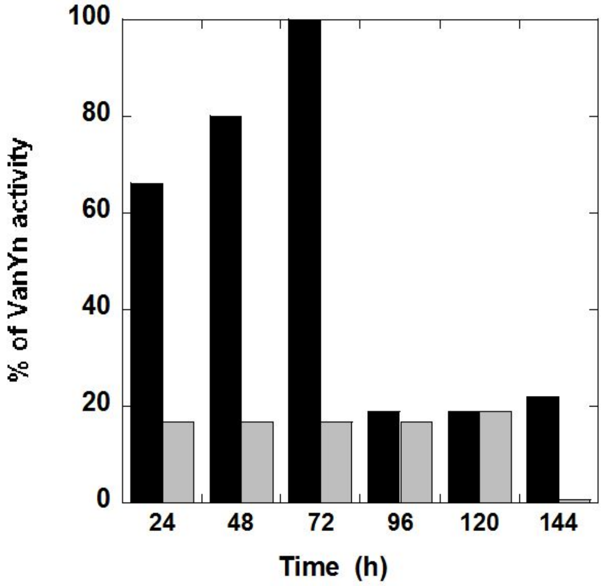

Figure S11. D,D-Carboxypeptidase activities in GPA-producing cultures of *N. gerenzanensis* in FM2 medium (black bars) and *N. coxensis* in ISP2lm medium (grey bars). Activity values were expressed as relative % of the maximum obtained at 72 h for *N. gerenzanensis* and were determined by measuring the amount of D-Ala released by hydrolysis of the *N*-acetyl-L-Lys-D-Ala-D-Ala tripeptide using a D-amino acid oxidase coupled to a peroxidase[14,15].

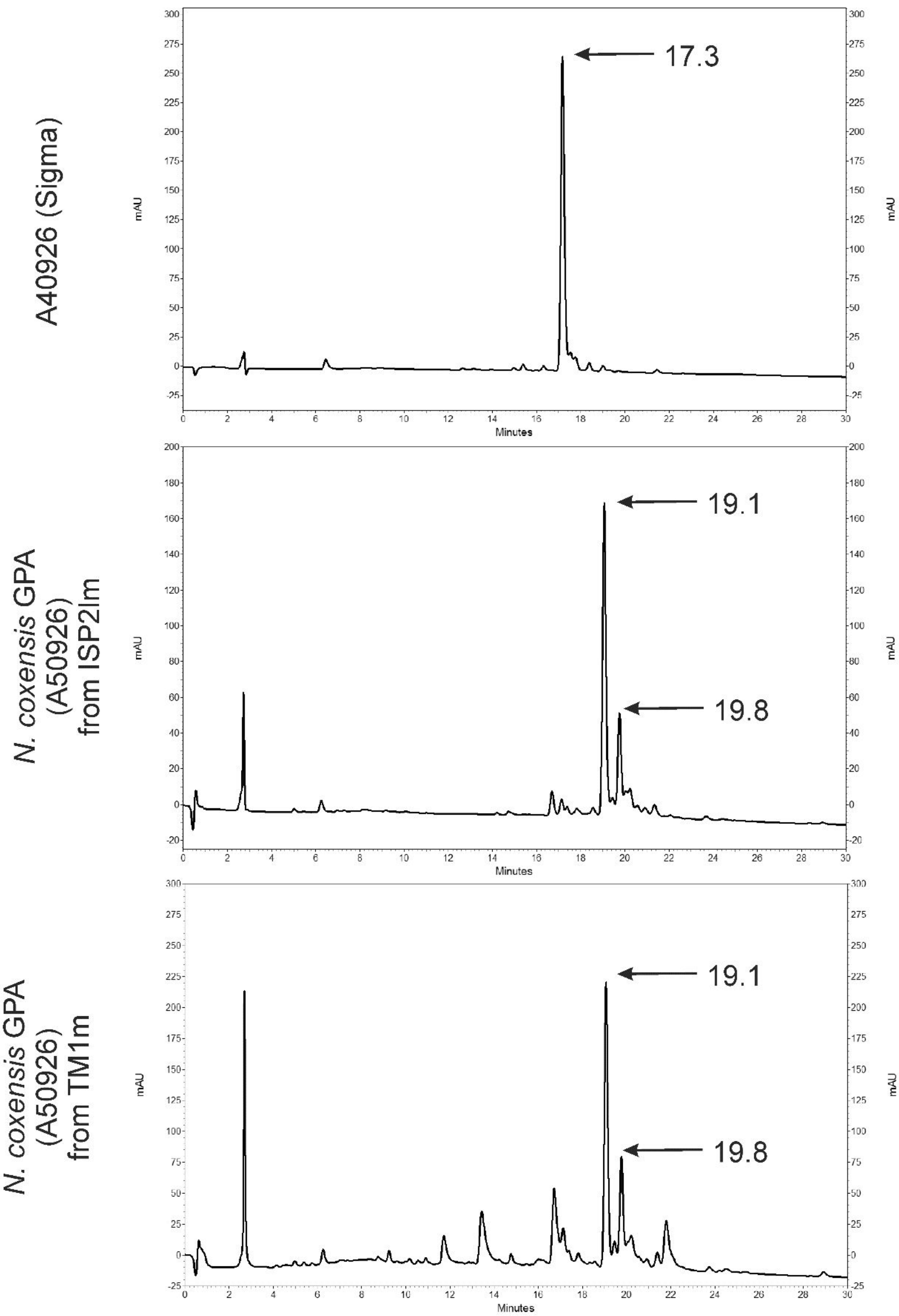

Figure S12. HPLC chromatograms with detection at 236 nm wavelength showing the partially purified GPA from *N. coxensis* (that we named A50926) cultivated for 168 h in either ISP2lm or TM1m. In both cases, the antibiotic complex contained two major peaks with retention times of 19.1 and 19.8 min, respectively. In the same HPLC conditions, the retention time of commercial A40926 standard (A40926 B) was 17.3 min.

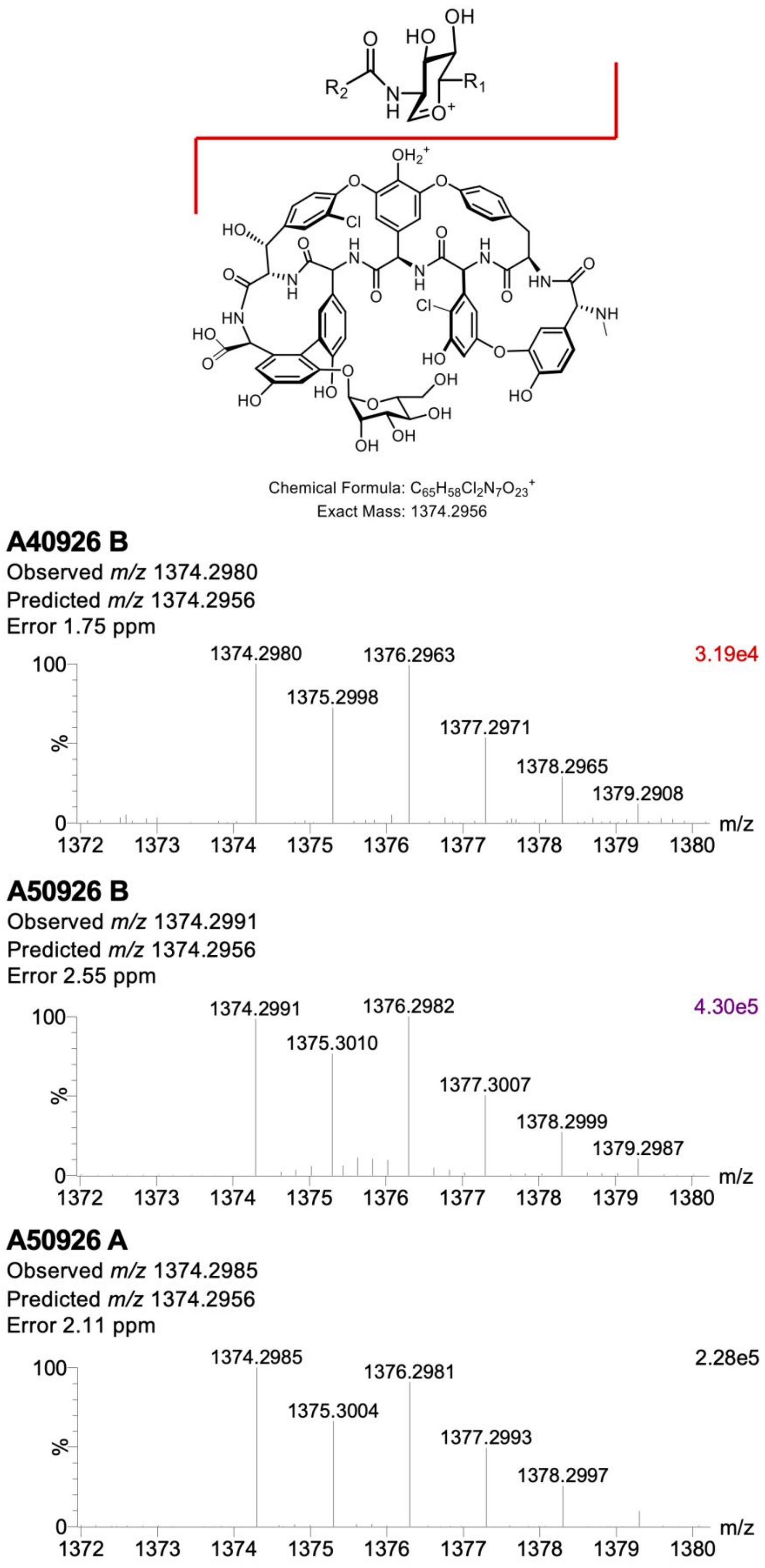

Figure S13. MS analysis of the in-source fragment corresponding to the mannosylated aglycone of the A40926 and A50926 GPAs. The schematic on top depicts the fragmentation leading to this peak, whereas the spectra below show masses and isotopic patterns for the fragment in all three molecules analyzed. Errors in ppm between the accurate observed masses of these fragments and the predicted mass are presented above each chromatogram. The intensity of the top peak in each spectrum is shown on the top right corner of each plot.

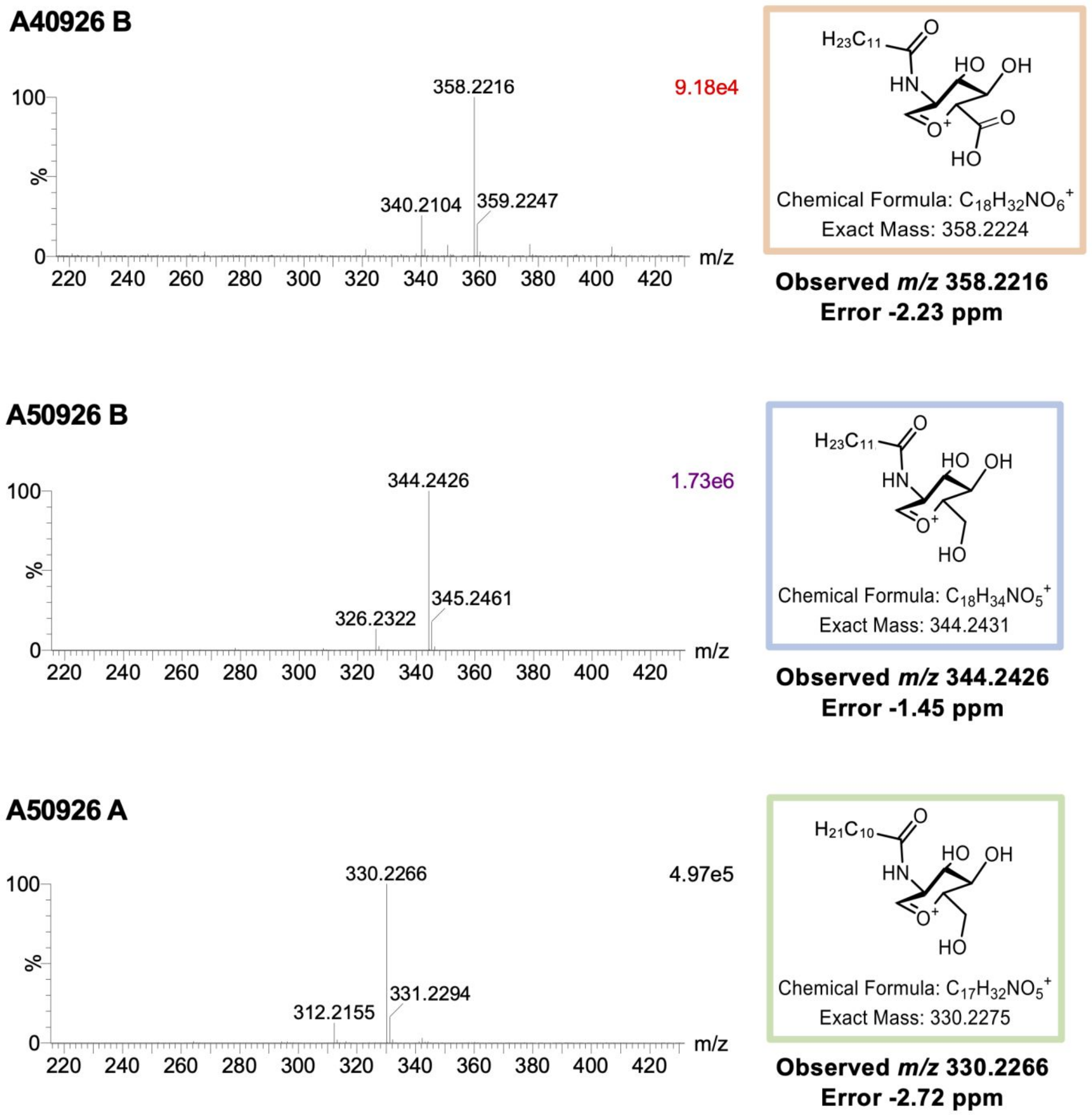

Figure S14. MS analysis of the in-source fragment corresponding to the GlcN-Acyl moieties of the A40926 and A50926 GPAs. The proposed structure for this moiety in each of the molecules analyzed is depicted next to its corresponding MS spectrum. Deviation in ppm between the accurate observed masses of these fragments and the predicted mass are presented below each proposed structure. The intensity of the top peak in each spectrum is shown on the top right corner of each plot.

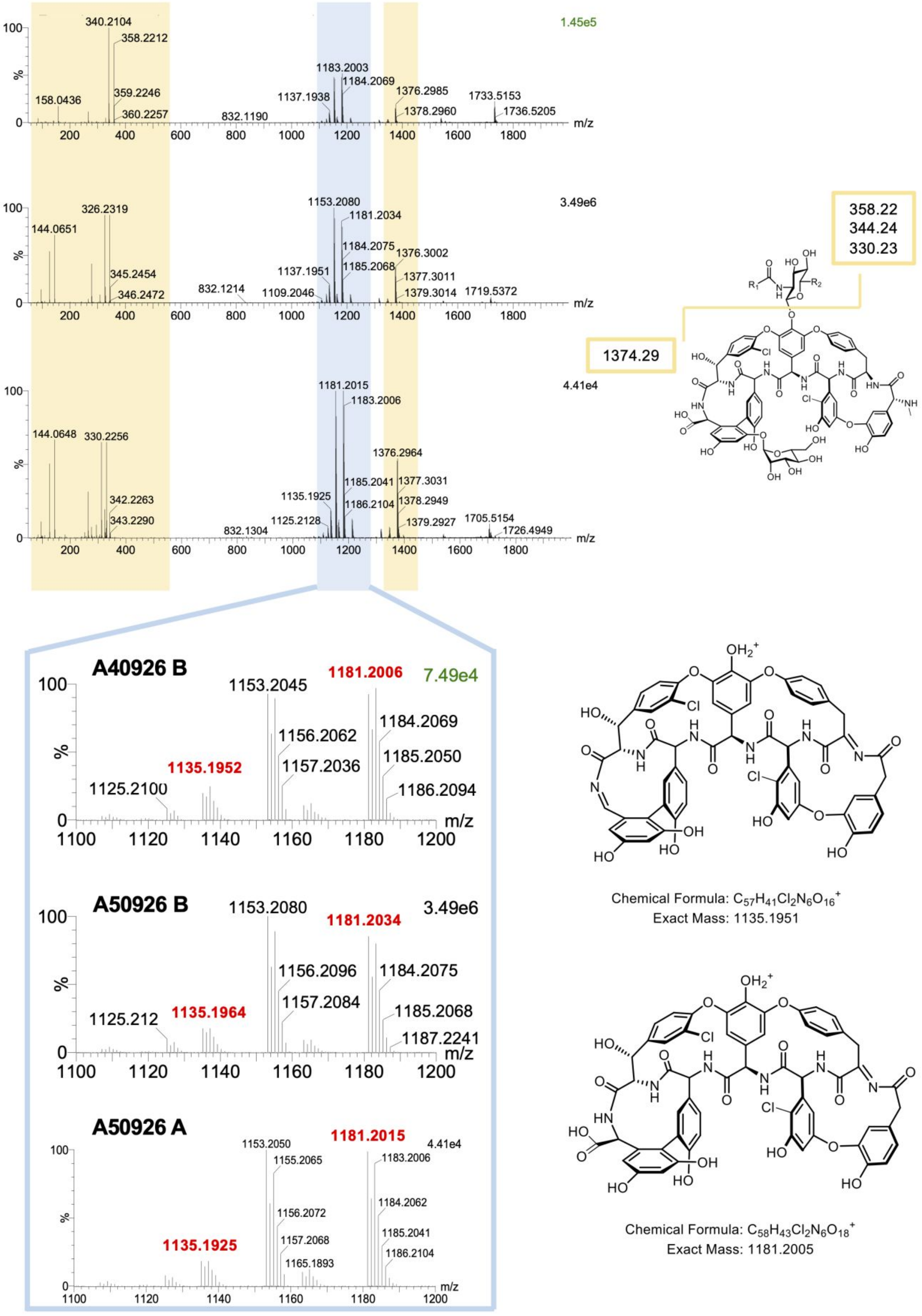

Figure S15. MS/MS analysis of A40926 B, A50926 B and A50926 A GPAs. Regions of the spectra corresponding to in-source fragments analyzed previously are highlighted in yellow, whereas a detailed view of aglycone fragmentation (highlighted in blue) is shown below. Monoisotopic masses of fragments for which a structure is proposed are highlighted in red in the spectra. Peak intensities are shown in the top right corner of each spectrum.

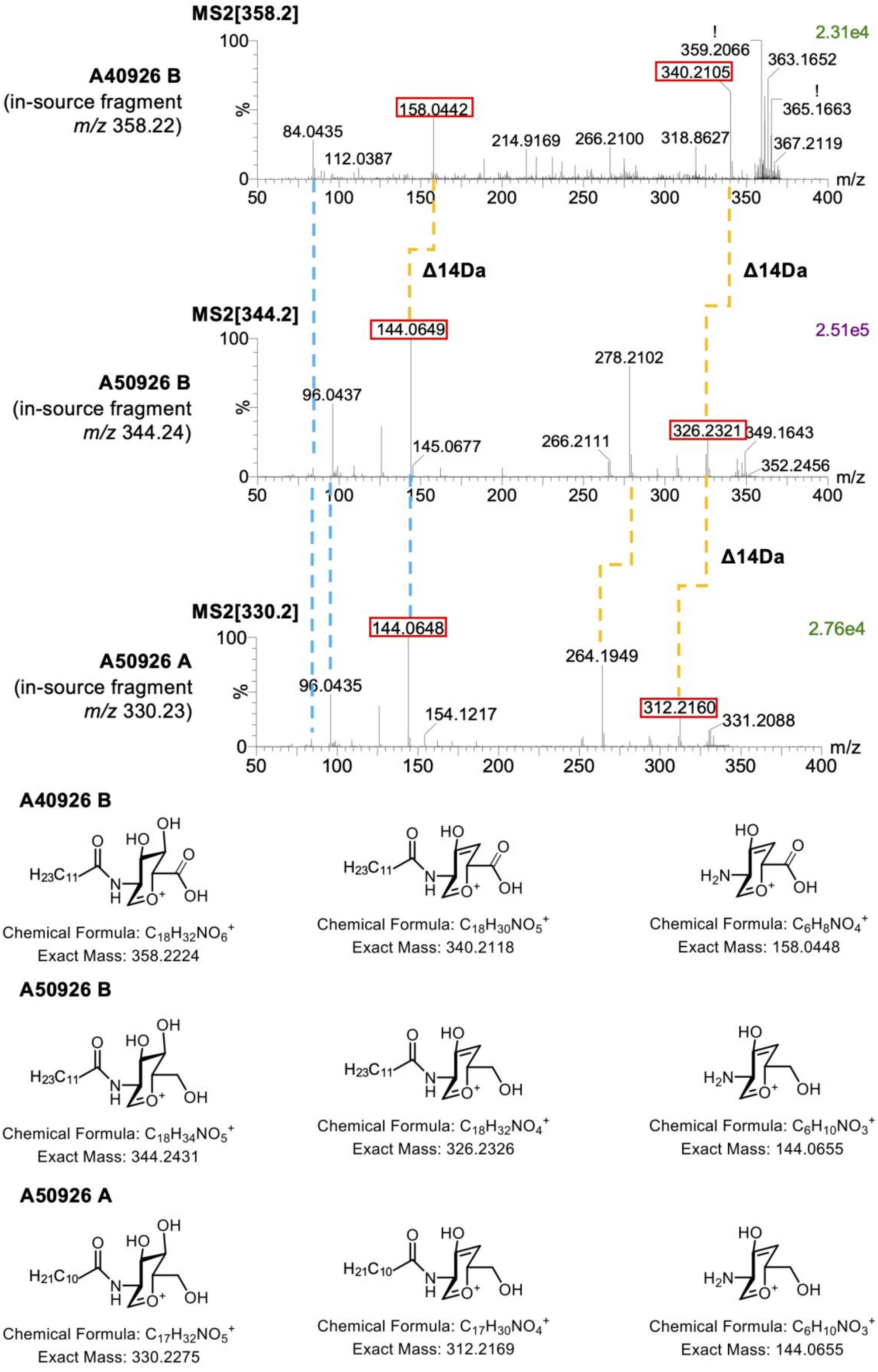

Figure S16. MS/MS analysis of the in-source signature fragment corresponding to the GlcN-Acyl moieties of the A40926 and A50926 GPAs. Identical fragments across several molecules are indicated with blue dashed lines, whereas fragments with distinctive mass losses are indicated with yellow dashed lines. Fragments for which proposed structures are presented in the bottom of the figure are circled in red in the MS/MS spectra. The intensity of the top peak in each spectrum is shown on the top right corner of each plot.

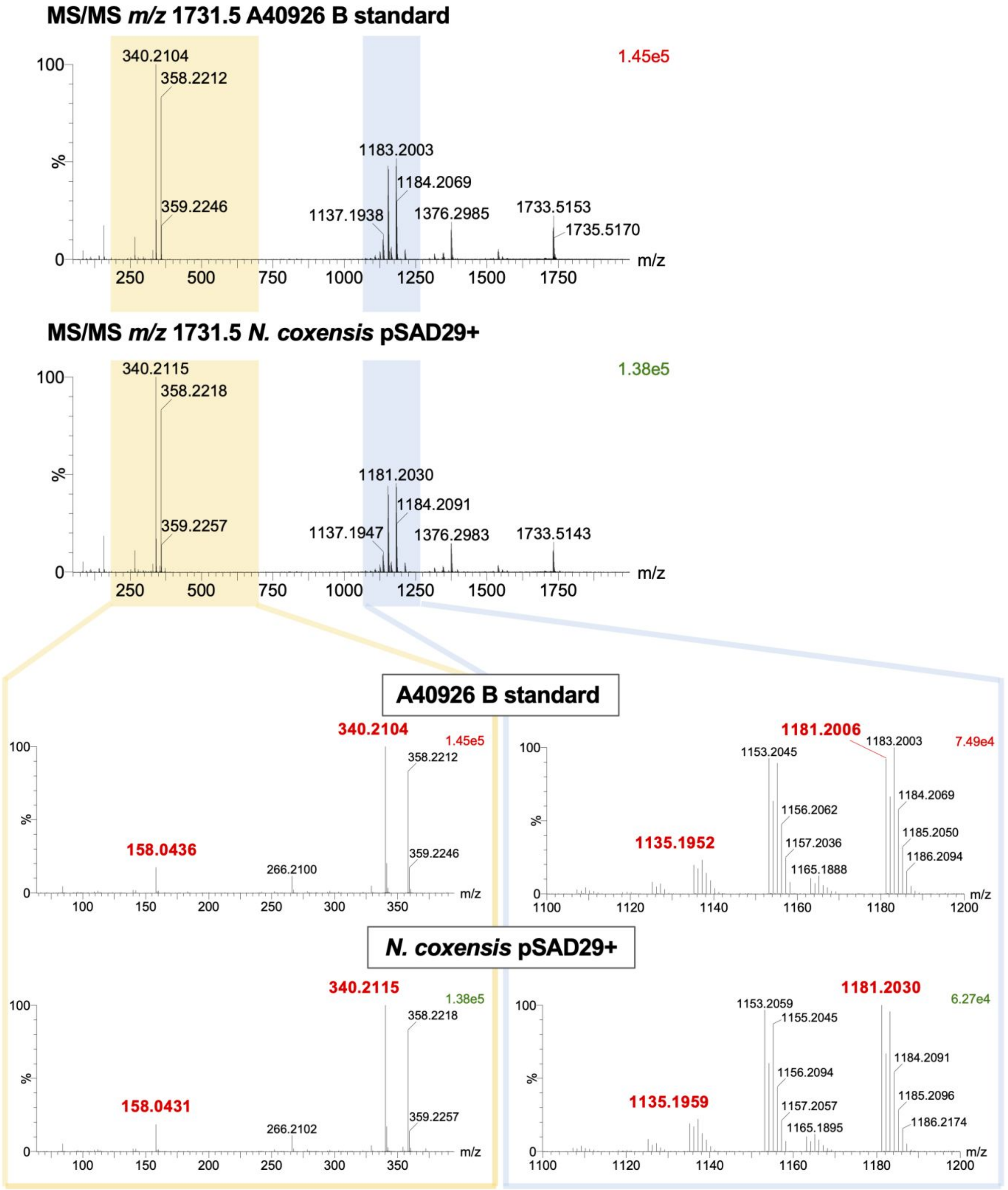

Figure S17. Comparison of the MS/MS spectra of a commercial standard of A40926 and A40926 produced by *N. coxensis* pSAD29+. The areas corresponding to the signature GlcN-Acyl moiety and aglycone fragments of the spectra are zoomed out at the bottom of the figure for clarity. Monoisotopic masses of fragments previously identified are highlighted in red and top peak intensities for each spectrum are shown in their top right corner.

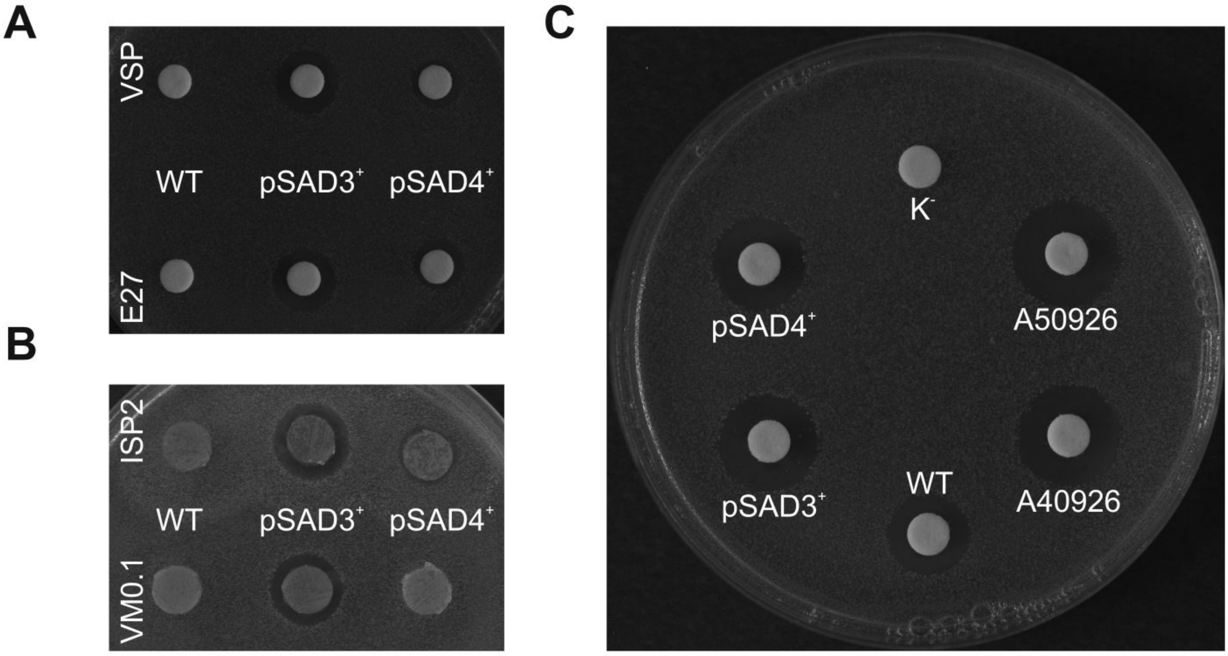

Figure S18. Antimicrobial activity assays towards *B. subtilis* ATCC 6633 showing: (A) activation of antimicrobial activity in recombinant strains overexpressing *dbv3* (pSAD3$^+$) and *dbv4* (pSAD4$^+$) cultivated in liquid VSP and E27 media, where the wild type does not produce any antimicrobial activity; (B) activation of the antimicrobial activity in solid media, where agar plugs cut from the lawns of *N. coxensis* pSAD3$^+$ grown on ISP2 and VM0.1 gave antimicrobial activity against *B. subtilis,* while the wild type and *N. coxensis* pSAD4$^+$ did not; (C) antimicrobial activity of borate buffer extracts from 168 h cultures of *N. coxensis* wild type and of recombinant strains pSAD3$^+$ and pSAD4$^+$ in ISP2lm compared with 1 µg of A40926 and 1 µg of partially purified A50926; K$^-$ was 50 µL of borate buffer as a control.

Supplementary references

(1) Kieser, T., Bibb, M. J., Buttner, M. J., Chater, K. F., Hopwood, D. A. Practical *Streptomyces* genetics. *The John Innes Foundation, Norwich, England*, 2000.

(2) Röttig, M., Medema, M. H., Blin, K., Weber, T., Rausch, C., Kohlbacher, O. NRPSpredictor2 – a web server for predicting NRPS adenylation domain specificity. *Nucleic Acids Res.*, 2011, *39*. https://doi.org/10.1093/nar/gkr323.

(3) Horbal, L., Kobylyanskyy, A., Yushchuk, O., Zaburannyi, N., Luzhetskyy, A., Ostash, B., Marinelli, F., Fedorenko, V. Evaluation of heterologous promoters for genetic analysis of *Actinoplanes teichomyceticus* - producer of teicoplanin, drug of last defense. *J. Biotechnol.*, 2013, *168*, 367–372. https://doi.org/10.1016/j.jbiotec.2013.10.018.

(4) Horbal, L., Kobylyanskyy, A., Truman, A. W., Zaburranyi, N., Ostash, B., Luzhetskyy, A., Marinelli, F., Fedorenko, V. The pathway-specific regulatory genes, *tei15** and *tei16**, are the master switches of teicoplanin production in *Actinoplanes teichomyceticus*. *Appl. Microbiol. Biotechnol.*, 2014, *98*, 9295–9309. https://doi.org/10.1007/s00253-014-5969-z.

(5) Yushchuk, O., Andreo-Vidal, A., Marcone, G. L., Bibb, M., Marinelli, F., Binda, E. New molecular tools for regulation and improvement of A40926 glycopeptide antibiotic production in *Nonomuraea gerenzanensis* ATCC 39727. *Front. Microbiol.*, 2020, *11*. https://doi.org/10.3389/fmicb.2020.00008.

(6) Nazari, B., Forneris, C. C., Gibson, M. I., Moon, K., Schramma, K. R., Seyedsayamdost, M. R. *Nonomuraea* sp. ATCC 55076 harbours the largest actinomycete chromosome to date and the kistamicin biosynthetic gene cluster. *MedChemComm*, 2017, *8*, 780–788. https://doi.org/10.1039/c6md00637j.

(7) Kumar, S., Stecher, G., Li, M., Knyaz, C., Tamura, K. MEGA X: molecular evolutionary genetics analysis across computing platforms. *Mol. Biol. Evol.*, 2018, *35*, 1547–1549. https://doi.org/10.1093/molbev/msy096.

(8) Darling, A. C. E., Mau, B., Blattner, F. R., Perna, N. T. Mauve: multiple alignment of conserved genomic sequence with rearrangements. *Genome Res.*, 2004, *14*, 1394–1403. https://doi.org/10.1101/gr.2289704.

(9) Cabanettes, F., Klopp, C. D-GENIES: dot plot large genomes in an interactive, efficient and simple way. *PeerJ*, 2018, *6,* e4958. https://doi.org/10.7717/peerj.4958.

(10) Sievers, F., Higgins, D. G. Clustal Omega. *Curr. Protoc. Bioinformatics*, 2014, *48*, 3.13.1-3.13.16. https://doi.org/10.1002/0471250953.bi0313s48.

(11) Alduina, R., Lo Piccolo, L., D'Alia, D., Ferraro, C., Gunnarsson, N., Donadio, S., Puglia, A. M. Phosphate-controlled regulator for the biosynthesis of the dalbavancin precursor A40926. *J. Bacteriol.*, 2007, *189*, 8120–8129. https://doi.org/10.1128/JB.01247-07.

(12) Pootoolal, J., Thomas, M. G., Marshall, C. G., Neu, J. M., Hubbard, B. K., Walsh, C. T., Wright, G. D. Assembling the glycopeptide antibiotic scaffold: the biosynthesis of A47934 from *Streptomyces toyocaensis* NRRL15009. *PNAS*, 2002, *99*, 8962–8967. https://doi.org/10.1073/pnas.102285099.

(13) Stachelhaus, T., Walsh, C. T. Mutational analysis of the epimerization domain in the initiation module PheATE of gramicidin S synthetase. *Biochemistry*, 2000, *39*, 5775–5787. https://doi.org/10.1021/bi9929002.

(14) Binda, E., Marcone, G. L., Pollegioni, L., Marinelli, F. Characterization of VanYn, a novel D,D-peptidase/D,D-carboxypeptidase involved in glycopeptide antibiotic resistance in

Nonomuraea sp. ATCC 39727. *FEBS Journal*, 2012, *279*, 3203–3213. https://doi.org/10.1111/j.1742-4658.2012.08706.x.

(15) Binda, E., Marcone, G. L., Berini, F., Pollegioni, L., Marinelli, F. *Streptomyces* spp. as efficient expression system for a D,D-peptidase/D,D-carboxypeptidase involved in glycopeptide antibiotic resistance. *BMC Biotechnol.*, 2013, *13*. https://doi.org/10.1186/1472-6750-13-24.
